# Lactic acidosis: implications for human exercise performance

**DOI:** 10.1007/s00421-025-05750-0

**Published:** 2025-03-15

**Authors:** Simeon P. Cairns, Michael I. Lindinger

**Affiliations:** 1 Sport and Recreation Research Institute New Zealand, School of Sport and Recreation, Faculty of Health and Environmental Sciences, Auckland University of Technology, Private Bag 92006, Auckland, 1020 New Zealand; 2 Health and Rehabilitation Research Institute, Faculty of Health and Environmental Sciences, Auckland University of Technology, Auckland, 1020 New Zealand; 3 Research and Development, The Nutraceutical Alliance Inc, Guelph, ON L8N 3Z5 Canada

**Keywords:** Lactate, Acidosis, Potassium, Inorganic phosphate, Skeletal muscle fatigue, Exercise performance

## Abstract

During high-intensity exercise a lactic-acidosis occurs with raised myoplasmic and plasma concentrations of lactate^−^ and protons ([lactate^−^], [H^+^] or pH). We critically evaluate whether this causes/contributes to fatigue during human exercise. Increases of [lactate^−^] per se (to 25 mM in plasma, 50 mM intracellularly) exert little detrimental effect on muscle performance while ingestion/infusion of lactate^−^ can be ergogenic. An exercise-induced intracellular acidosis at the whole-muscle level (pH_i_ falls from 7.1–7.0 to 6.9–6.3), incorporates small changes in slow-twitch fibres (pH_i_ ~ 6.9) and large changes in fast-twitch fibres (pH_i_ ~ 6.2). The relationship between peak force/power and acidosis during fatiguing contractions varies across exercise regimes implying that acidosis is not the sole cause of fatigue. Concomitant changes of other putative fatigue factors include phosphate metabolites, glycogen, ions and reactive oxygen species. Acidosis to pH_i_ 6.7–6.6 at physiological temperatures (during recovery from exercise or induced in non-fatigued muscle), has minimal effect on force/power. Acidosis to pH_i_ ~ 6.5–6.2 per se reduces maximum force (~12%), slows shortening velocity (~5%), and lowers peak power (~22%) in non-fatigued muscles/individuals. A pre-exercise induced-acidosis with ammonium chloride impairs exercise performance in humans and accelerates the decline of force/power (15–40% initial) in animal muscles stimulated repeatedly in situ. Raised [H^+^]_i_ and diprotonated inorganic phosphate ([H_2_PO_4_^−^]_i_) act on myofilament proteins to reduce maximum cross-bridge activity, Ca^2+^-sensitivity, and myosin ATPase activity. Acidosis/[lactate^−^]_o_ attenuates detrimental effects of large K^+^-disturbances on action potentials and force in non-fatigued muscle. We propose that depressive effects of acidosis and [H_2_PO_4_^−^]_i_ on myofilament function dominate over the protective effects of acidosis/lactate^−^ on action potentials during fatigue. Raised extracellular [H^+^]/[lactate^−^] do not usually cause central fatigue but do contribute to elevated perceived exertion and fatigue sensations by activating group III/IV muscle afferents. Modulation of H^+^/lactate^−^ regulation (via extracellular H^+^-buffers, monocarboxylate transporters, carbonic anhydrase, carnosine) supports a role for intracellular acidosis in fatigue. In conclusion, current evidence advocates that severe acidosis in fast-twitch fibres can contribute to force/power fatigue during intense human exercise.

**Supplementary Information:**

The online version contains supplementary material available at 10.1007/s00421-025-05750-0.

## Introduction

It has long been postulated that lactic acid is a harmful chemical formed in working skeletal muscle that impairs exercise performance. This notion has become known as the “lactic acid hypothesis of fatigue”. During intense exercise of more than a brief duration there is an acute decline of muscle or exercise performance defined as fatigue (Allen et al. 2008; Cairns 2013; Knicker et al. 2011). The relationships between fatiguing exercise, lactic acid, and acidosis in humans were first described more than 100 years ago (Fletcher and Hopkins 1907; Hill and Lupton 1923), with the early research well summarised in Jervell’s thesis (Jervell 1928). To this day there remains a strong belief amongst exercise and sport physiologists, athletes and coaches, that lactic acidosis is the major villain underpinning fatigue. Despite this, a fundamental scientific point is that virtually no lactic acid appears in the body during exercise (Lindinger et al. 2005; Robergs et al. 2004). Rather lactic acid exists as two ionic species, namely lactate anions (lactate^−^) and hydrogen ions/protons (H^+^). Although the latter is, in reality, hydronium (H_3_O^+^) ions, it is conventional to represent it as H^+^ and measure it as pH (pH = −log_10_[H^+^]). With contracting muscle, it is necessary to evaluate intra- and extracellular lactate^−^ and H^+^ as potential factors in fatigue since these changes typically occur together and often when there is a decline of performance. Hence, lactic acidosis has long been touted as a mechanism of fatigue (Fletcher and Hopkins 1907; Hill and Lupton 1923; Jervell 1928). Notably, association does not mean direct cause or even indirect contribution. In fact, such associative correlations between muscle fatigue and lactic acidosis have led to erroneous cause-effect conclusions.

Despite the continued entrenchment in our psyche that lactate^−^ and acidosis are bad end-products of metabolism, it became apparent in the 1990s and early 21st Century, that there are weighty challenges to the lactic acid hypothesis. In consequence, the 2006 *Sports Medicine* review “Lactic acid and exercise performance: culprit or friend” endeavored to present the then current and contrasting scientific findings in a balanced manner whereby readers could critically evaluate the roles of both ions in fatigue (Cairns 2006). About that time the *Journal of Applied Physiology* hosted a point-counterpoint debate that “Lactic acid accumulation is an advantage/disadvantage during muscle activities” where authors provided their arguments, in a polarised manner, either for or against a role of lactate^−^ and/or acidosis in fatigue (Bangsbo and Juel 2006; Lamb and Stephenson 2006). This incited debate but was necessarily without consensus. Ten years later in *Medicine and Science in Sports and Exercise* a strong pro-perspective favoring acidosis as a major cause of fatigue was argued by Fitts (2016), whereas the opposing view was asserted by Westerblad (2016). Clearly, the importance of H^+^/lactate^−^ in fatigue remained unresolved. In recent times several reviews have provided further detail on the physiological roles of aspects of these two ions (Brooks 2018; Brooks et al. 2022, 2023; Debold et al. 2016; Ferguson et al. 2018; Hostrup et al. 2021; Sundberg and Fitts 2019). From the early 21st Century until now, some impressive advances with superb experiments have generated new data addressing the roles of H^+^/lactate^−^ as players in fatigue and these studies will be discussed in the present review.

## Perspectives by the end of the 20th century

**Fig. 1:**
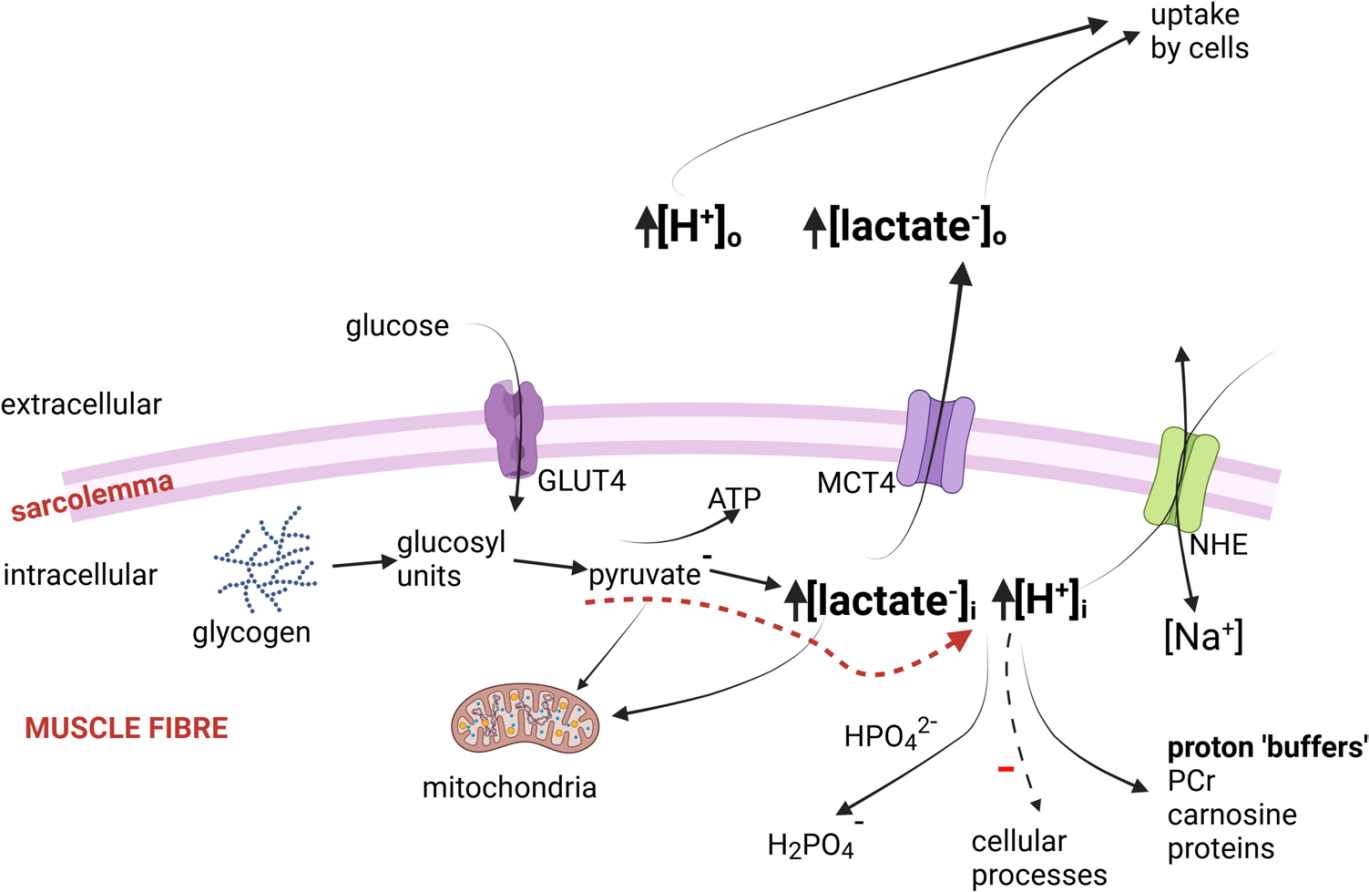
Schematic presentation that depicts how lactate^−^ and H^+^ are produced within a muscle fibre and then extruded into extracellular fluids. Glycogen and glucose are substrates giving rise to formation of H^+^/lactate^−^. Lactate^−^ is translocated across the sarcolemma via monocarboxylate lactate transporters (e.g. MCT4) or sequestered into mitochondria. The increased [H^+^]_i_ is buffered, binds to inorganic phosphate forming diprotonated phosphate (H_2_PO4^−^), and influences cellular processes. [H^+^]_i_ is regulated by changing the concentrations of strong and weak ions within the cell, sometimes in association with MCT4 or Na^+^-H^+^ exchanger (NHE) activity. Created using Biorender

During repeated muscle contractions the need to generate and maintain adenosine triphosphate (ATP) requires activation of glycogenolysis and glycolysis, whether under aerobic or anaerobic conditions. When the demand for ATP cannot be met from phosphocreatine (PCr) hydrolysis, which is limited, then glucosyl units derived from either muscle glycogen or glucose transported into the muscle fibre (via GLUT4) are utilised in glycogenolytic/glycolytic reactions. Lactate^−^ is formed intracellularly from glycolytic reactions together with the stoichiometric production of H^+^ (Fig. 1). The increase of [H^+^]_i_ is attributed to ionic interactions within intracellular fluid (Kowalchuk et al. 1988a; Lindinger et al. 2005; Stewart 1983 and/or associated biochemical reactions (Robergs et al. 2004). Lactate^−^ by virtue of being a strong acid anion accounts for up to 50% of the acidosis within muscle fibres during exercise (Heigenhauser and Lindinger 1988; Kowalchuk et al. 1988a; Stewart 1983). A lactic acidosis during intense exercise or ischemic muscle contractions simply describes that the concentrations of lactate^−^ and H^+^ have increased, with there being evidence for a close association between these two ions (Kemp et al. 2001; Marcinek et al. 2010; Sahlin et al. 1975, 1976). Raised [H^+^]/[lactate^−^] in the myoplasm of contracting muscle fibres then leads to increases within the transverse (T-) tubular system, interstitial fluid, and venous plasma, following lactate^−^ extrusion across the sarcolemma. Lactate^−^ efflux from the fibre occurs primarily via monocarboxylate transporters (MCT), with the sodium-hydrogen exchanger (NHE) acting to maintain charge balance (Fig. 1).

Given this background we now highlight several key points identified as being for or against H^+^/lactate^−^ as factors underpinning fatigue (Bangsbo and Juel 2006; Cairns 2006; Lamb and Stephenson 2006). In support: increases of [H^+^]/[lactate^−^] occur in fatiguing muscle during intense exercise, together with a decline of muscle force; an induced acidosis can reduce muscle force/power in resting and fatiguing muscles of animals; mechanisms are available to explain how increased [H^+^]_i_ reduces force at room temperature; and exogenous application of H^+^-buffers, such as sodium bicarbonate (NaHCO_3_), can improve performance during intense exercise. In opposition: force changes during fatiguing exercise and recovery are often not temporally aligned with changes of pH_i_; an induced acidosis protects against the loss of isometric force at raised extracellular [K^+^], ([K^+^]_o_) in vitro, noting that H^+^- and K^+^-disturbances occur concomitantly during intense exercise; and force depressing effects of induced acidosis in non-fatigued muscle are attenuated at higher more physiological temperatures.

### Purpose of the present review

We aim to synthesize the findings of the past 20 years of research, along with historical research, to provide a coherent state-of-the-art evaluation on the roles of H^+^/lactate^−^ in human exercise performance. To do this we address four main questions: (i) What are the [lactate^−^] and pH (or [H^+^]) values achieved in myoplasm and plasma during various high-intensity exercise regimes? Also, what other factors change concomitantly during such exercise that interact with H^+^/lactate^−^ or the physiological processes they affect? (ii) Does H^+^/lactate^−^ (with interacting factors) cause protection or impairment of muscle or exercise performance? (iii) Are there authentic mechanisms (peripheral or central) that explain impairment of performance with H^+^/lactate^−^ under physiological conditions? (iv) Does selective manipulation of H^+^/lactate^−^ regulatory processes have any influence on fatigue? To answer these questions, we intentionally focus on studies involving muscle or exercise performance in humans whenever possible.

Many experimental approaches have been used to address these questions which in turn has led to debate over which results are the most physiologically relevant to the in vivo situation. Here we evaluate research findings involving human exercise (or fatigue models), electrical stimulation models in situ or in vitro using intact whole muscles, fibre bundles, or isolated single fibres, and from preparations such as skinned muscle fibres (where the cell membrane has been chemically or mechanically removed), isolated sarcoplasmic reticulum (SR), or isolated proteins e.g., myosin. While the latter reduced muscle preparations are certainly needed to investigate mechanisms, we take the approach of trying to translate the results of this research into the intact human. We discuss effects on human muscle, or mammalian rather than amphibian muscle, and mainly refer to studies with temperatures exceeding 20 °C, unless stated otherwise.

## Changes of intra- and extracellular lactate^−^ and pH with high-intensity exercise

Lactate^−^ and pH can readily be measured intracellularly within muscle fibres, and extracellularly in venous or arterial blood, under resting conditions, and during or after exercise. Such measurements have only sometimes been made in the interstitium (MacLean et al. 2000; Street et al. 2005) or T-system lumen (Launikonis et al. 2018). The [lactate^−^] and pH (or [H^+^]) values obtained from human muscles immediately after high-intensity exercise, simulated sports, or muscle stimulation models are presented in Tables 1 and 2, respectively. Studies of large muscle groups have traditionally measured [lactate^−^]_i_ and pH_i_ chemically in muscle homogenates with samples obtained using biopsy, which is somewhat delayed post-exercise (10–60 s). Phosphorus nuclear magnetic resonance spectroscopy (^31^P-MRS) is nowadays routinely employed for continuous assessment of pH_i_ (and phosphate metabolites). This technique has evolved from using only small muscles to entire limbs, and now whole-body, with isometric or dynamic contractions, and with some ability to discriminate between muscle fibre-type responses. A crucial point is that in fatigued muscle the absolute pH_i_ (or [H^+^]_i_) needs to be shown, rather than change of pH_i_, (or % change) since it is absolute pH_i_ levels that affect muscle cellular processes.

**Table 1:** Lactate^−^ concentrations within human muscle fibres and in venous blood, at rest and the end of high-intensity exercise of 30 s to 10 min duration

Exercise event	[lactate^−^]_i_ (mM)	[lactate^−^]_o_ (mM)
Resting	1.6 (0.4–3.4)	1.2 (0.5–3.8)
	(*n* = 31)	(*n* = 41)
Simulated sports		
Running (legs)	21.6 (9.9–31.4)	12.4 (9.8–16.4)
	(*n* = 8)	(*n* = 9)
Cycling (legs)	35.9 (27.2–51.6)	13.2 (7.0–22.0)
	(*n* = 16)	(*n* = 19)
Rowing	–	19.6 (16.2–26.2)
		(*n* = 8)
Exercise models		
Repeated contractions (legs)	33.6 (20.7–41.0)	10.7 (8.0–14.1)
	(*n* = 4)	(*n* = 4)
Repeated contractions (arms)	–	5.8 (5.0–7.1)
		(*n* = 5)
Continuous static contractions (legs)	29.3 (20.3–37.3)	–
	(*n* = 6)	
Stimulation models		
Leg contractions	34.7 (26.4–50.9)	–
	(*n* = 5)	

Data are the average of mean values across studies, range of mean values is shown in brackets, *n* = number of studies. High-intensity exercise refers to dynamic exercise at VO_2_peak or greater, incremental dynamic exercise to exhaustion, repeated or sustained maximum voluntary isometric contractions. [lactate^−^]_i_ = intramuscular lactate^−^ concentration determined from leg muscle (quadriceps, calf) biopsies. End-exercise/stimulation values were obtained soon after exercise cessation. Lactate^−^ concentrations expressed per dry or wet weight were converted to mmol/L H_2_O (Kemp et al. 2007; Kowalchuk et al. 1988a, b. [lactate^−^]_o_ = plasma venous lactate^−^ concentration. Simulated sports involved whole-body exercise on ergometers. Exercise models involved voluntary contractions of single muscle groups. Simulation models involved intermittent/continuous electrical stimulation of muscle via nerve or muscle membranes, sometimes with blood flow being occluded. Studies used are in Supplementary File 1

**Table 2:** pH or [H^+^] values within human muscle fibres and in venous blood, at rest and the end of high-intensity exercise of 30 s to 10 min duration

Exercise event	pH_i_	[H^+^]_i_ (nM)	pH_o_
Resting	7.06 (6.88–7.30)	88 (50–132)	7.40 (7.35–7.45)
	(*n* = 78)		(*n* = 40)
Simulated sports			
Running (legs)	6.80 (6.63–6.92)	162 (120–234)	7.14 (7.07–7.25)
	(*n* = 8)		(*n* = 9)
Cycling (legs)	6.61 (6.40–6.81)	256 (155–398)	7.13 (6.95–7.25)
	(*n* = 21)		(*n* = 15)
Rowing (arms)	6.31 (6.30–6.32)	490 (479–50)	7.02 (6.85–7.20)
	(*n* = 2)		(*n* = 7)
Exercise models			
Repeated contractions (legs)	6.63 (6.40–6.90)	242 (125–398)	7.10 (7.07–7.13)
	(*n* = 21)		(*n* = 3)
Repeated contractions (arms)	6.38 (5.86–6.61)	466 (245–1202)	7.25 (7.20–7.29)
	(*n* = 20)		(*n* = 6)
Continuous static contractions (legs)	6.64 (6.47–6.89)	242 (129–339)	–
	(*n* = 9)		
Continuous static contractions (arms)	6.46 (6.34–6.58)	357 (263–457)	–
	(*n* = 4)		
Stimulation models			
Leg contractions	6.61 (6.43–6.70)	256 (200–372)	–
	(*n* = 4)		

Data are average of mean values across studies, range of mean values is shown in brackets, *n* = number of studies. High-intensity exercise refers to dynamic exercise at VO_2_peak or greater, incremental dynamic exercise to exhaustion, repeated or sustained maximum voluntary isometric contraction. pH_i_ = -log_10_[H^+^]_i_ = intramuscular pH; [H^+^]_i_ = intramuscular proton concentration; pH_o_ = plasma venous pH. End-exercise/stimulation values were obtained immediately at (^31^P-MRS) or soon after (biopsy homogenate) exercise cessation. Simulated sports involved whole-body exercise on ergometers. Exercise models involved voluntary contractions of single muscle groups. Simulation models involved intermittent/continuous electrical stimulation of muscle via nerve or muscle membranes, sometime with blood flow occluded. There was no significant difference between knee extensor (vastus lateralis) and calf (gastrocnemius, tibialus anterior) muscle data, hence these values were pooled to represent leg muscles. Studies used are in Supplementary File 2

When men performed repeated supramaximal isokinetic cycling (>300% VO_2_peak) for 30 s, the peak power decreased to 45% of maximal, whilst quadriceps [lactate^−^]_i_ increased to 47 mM, femoral venous [lactate^−^]_o_ increased to 13 mM, pH_i_ fell from 6.88 to 6.48 (or [H^+^]_i_, 328 nM), and venous pH_o_ decreased from 7.38 to 7.00 (Kowalchuk et al. 1988a, McCartney et al. 1986, see Fig. 5). These data corroborate that increased [lactate^−^] and decreased pH, occur together with a loss of power, but this does not necessitate that these ions are responsible for fatigue. Across many studies involving intense exercise the [lactate^−^]_i_ became elevated to 20–35 mM, with the highest value being ~50 mM (Table 1). Plasma venous [lactate^−^]_o_ often increases in the first several minutes post-exercise (by ~3–9 mM) (Costill et al. 1983; Harmer et al. 2000; Kowalchuk et al. 1988b) but we show end-exercise values (Tables 1 and 2) which align with contractile measurements. Minor decreases of pH_i_ from resting values of 7.1–7.0 to 7.0–6.8 occur during brief single or repeated sprints (e.g., <20 s) (Bishop et al. 2004; Bogdanis et al. 1998), prolonged or low-to-moderate exercise intensities (Churchward-Venne et al. 2010; Newham and Cady 1990; Stephens et al. 2002), including exercise below the critical power level (Jones et al. 2008), and in many team-game sports e.g., soccer, ice-hockey (Krustrup et al. 2006; Vigh-Larsen et al. 2020). Such small levels of myoplasmic acidosis do not depress force (Jubrias et al. 2003).

The muscle acidosis occurring during high-intensity simulated sports, exercise models with single muscle groups, and electrical stimulation of muscles for 30 s to 15 min is shown in Table 2. The mean pH_i_ for leg muscles (e.g. quadriceps, gastrocnemius, tibialis anterior) falls to ~6.6 (range of mean values 6.9–6.4) (e.g. Black et al. 2017; Broxterman et al. 2017a; Costill et al. 1983; Hermansen and Osnes 1972; Sundberg et al. 2019; Vigh-Larsen et al. 2022) with acidosis being significantly greater for wrist/finger muscles where pH_i_ falls to ~6.4 (range of mean values 6.6–5.9) (e.g. Newham and Cady 1990; Raymer et al. 2004; Taylor et al. 1983; Volianitis et al. 2018; Wilson et al. 1988). This difference may have arisen because of a greater buffer capacity in the quadriceps than arm muscles (Kemp et al. 2001). More recent pH_i_ and [lactate^−^] data have emerged for aged people (Arieta et al. 2024; Kent-Braun et al. 2002; Sundberg et al. 2019), different ethnic groups (Bret et al. 2013), elite athletes (Mildenhall et al. 2023), and between gender (Kent-Braun et al. 2002). Exercise-induced acidosis is often 0.1 pH units less in older than younger individuals (Arieta et al. 2024) although this is not always the case (Sundberg et al. 2019, 2024). Elite athletes generate huge increases in plasma [lactate^−^] sometimes exceeding 25 mM (Mildenhall et al. 2023; Nielsen 1999) with these individuals seldom permitting biopsies so that their muscle pH_i_ is uncertain. Trained athletes show a lesser muscle acidosis during constant-workload exercise along with greater abundance of H^+^-regulatory proteins including MCT, carbonic anhydrase, and NHE (Gunnarsson et al. 2013; Juel et al. 2004; Messonnier et al. 2007; Skattebo et al. 2024).

Linear correlations have been described for end-exercise pH_i_ and muscle fibre-type composition (%) for the wrist flexors (Mizuno et al. 1994) and quadriceps muscle (Mannion et al. 1995). This indicates that having a greater percentage of fast-twitch than slow-twitch fibres evoke a larger whole-muscle acidosis. Several ^31^P-MRS studies involving repeated contractions of human gastrocnemius, tibialis anterior, finger or wrist muscles have revealed compartments for pH_i_ that are likely to represent populations of slow-twitch or fast-twitch fibres (Achten et al. 1990; Mizuno et al. 1994; Park et al. 1987; Taylor et al. 1983; Vandenborne et al. 1991). According to these studies, pH_i_ fell in slow-twitch fibres to ~6.9 (range of mean values 7.1–6.8) and in fast-twitch fibres to ~6.2 (range of mean values 6.3–6.0). Hence, the largest realistic acidosis is to pH_i_ ~ 6.0 in fast-twitch fibres. Similarly, with animal muscles stimulated repeatedly in vitro or in situ, an intracellular acidosis occurs (to pH_i_ 6.9–6.2) which is more excessive in fast-twitch than slow-twitch muscle (Adams et al. 1991; Juel 1988a; Lindinger and Heigenhauser 1991; Liu et al. 2007). We recommend that future research should focus more on fast-twitch than slow-twitch fibres where a larger acidosis occurs.

### Time-course studies

**Fig. 2:**
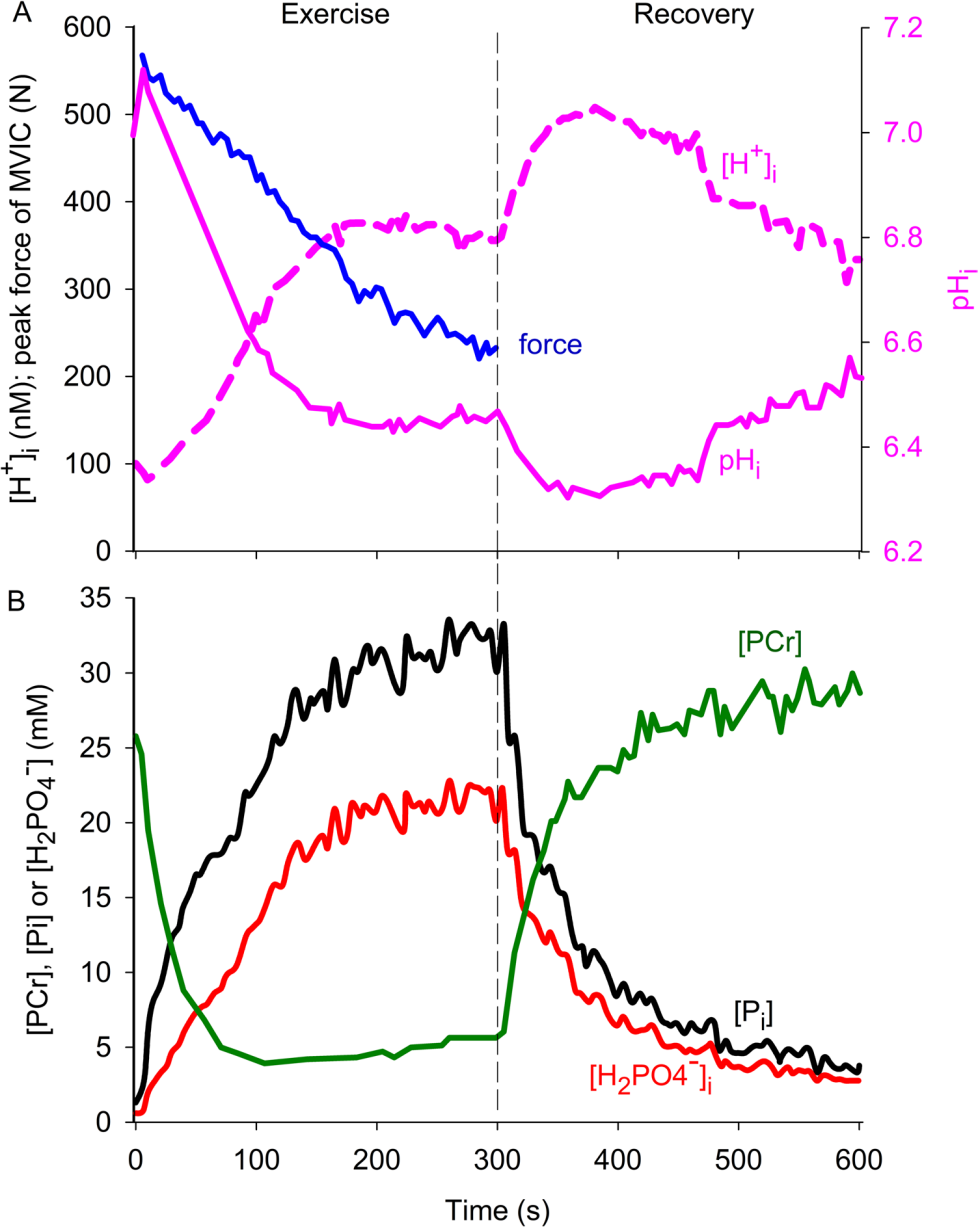
Continuous recordings of **A** peak maximum voluntary isometric contraction (MVIC) force, pH_i_, and [H^+^]_i_ and **B** concentrations of phosphocreatine, [PCr]_i_, total inorganic phosphate, [P_i_]_i_, and diprotonated phosphate, [H_2_PO_4_^−^]_i_, during fatigue and recovery of single leg knee-extensor muscles. The exercise model involved 60 repeated MVIC (3-s contraction, 2-s rest) over 5 min, followed by 5 min rest recovery. Recovery force data not available. Metabolic changes were assayed using magnetic resonance spectroscopy, i.e., ^31^P-MRS. Data from Broxterman et al. (2017a). Created using Biorender

Greater appraisal of the role for pH_i_ in fatigue can be obtained using continuous time-course measurements of force/power and pH_i_ throughout exercise and recovery. Figure 2 shows recordings of A) peak maximal voluntary isometric contraction (MVIC) force and pH_i_ (or [H^+^]_i_), and B) [PCr], total inorganic phosphate [P_i_] and diprotonated phosphate ([H_2_PO_4_^−^]), for the leg extensor muscles during and after repeated intermittent MVIC, that depressed peak force to 38% initial (Broxterman et al. 2017a). An alkalinisation of ~0.1 pH units, from the resting value of 7.0, appeared during the first 15 s of exercise at a time when force had fallen by ~10%. This effect is attributed to PCr hydrolysis with H^+^ consumption (Allen et al. 2008; Kemp et al. 2001; Sundberg and Fitts 2019). The pH_i_ then declined progressively (i.e. [H^+^]_i_ increased) as exercise proceeded towards a steady pH_i_ of ~6.45 ([H^+^]_i_, 355 nM). There is a time-dependence for development of intracellular acidosis which typically becomes maximal over 2–5 min exercise (Bartlett et al. 2020; Broxterman et al. 2018; Kent-Braun 1999; Miller et al. 1988; Newham and Cady 1990; Sundberg et al. 2019). In line with this, it appears that the glycogenolytic and glycolytic rates reach a steady level as exercise proceeds (Kemp et al. 2001). Immediately post-exercise, an abrupt further acidosis of 0.10–0.15 pH units transpires (Fig. 2A) when force is recovering. This is explained by H^+^ release associated with PCr resynthesis (Allen et al. 2008; Sundberg and Fitts 2019). The PCr, P_i_ and H_2_PO_4_^−^ recovered rapidly (Fig. 2B) and much faster than pH_i_. This single-leg exercise model clearly evoked an intracellular acidosis comparable to that achieved with intense whole-body locomotor sports (Table 2). Temporal dissociation between changes of pH_i_ and force occurs (i.e. early alkalinisation when force declines, steady pH_i_ yet force decreases further in late exercise, greater acidosis during early force recovery) which implies that other factors contribute to fatigue.

#### Summary

During high-intensity exercise the plasma [lactate^−^] can increase in extreme cases to 20–25 mM, pH_o_ falls to 7.0–6.9, and myoplasmic [lactate^−^] rises to 25–50 mM. Importantly, pH_i_ can fall to 6.3–6.0 in fast-twitch fibres, whilst there is only a minor acidosis in slow-twitch fibres.

## Relationships between peak force/power and pH_i_ (or [H^+^]_i_) during fatiguing contractions

**Fig. 3:**
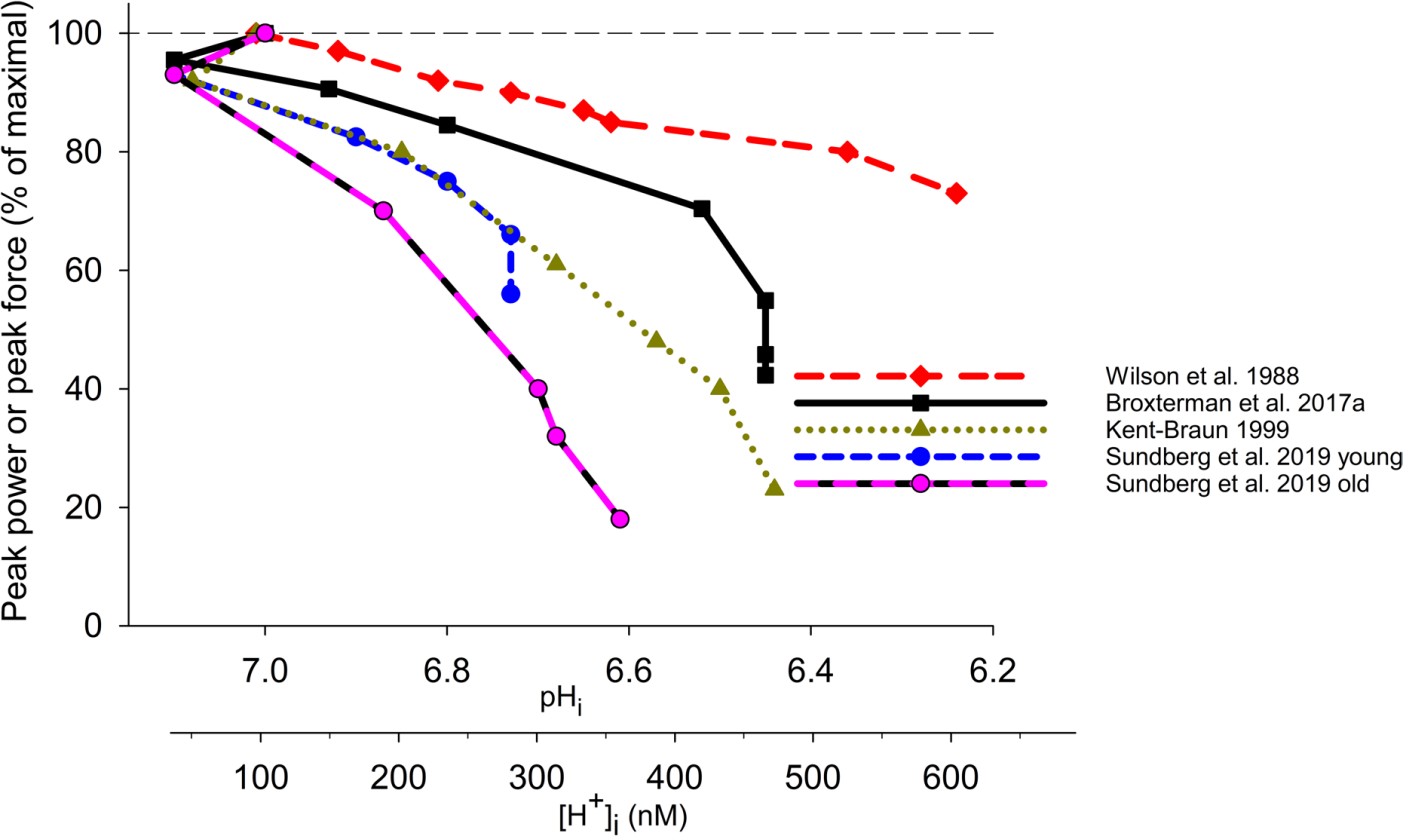
Relationships between peak force/power and pH_i_ or [H^+^]_i_ recorded continuously in human muscles during fatiguing contractions. pH_i_ determined by ^31^P-MRS. Contractile measures were peak force (Broxterman et al. 2017a; Kent-Braun 1999; Wilson et al. 1988) and peak power (Sundberg et al. 2019). Exercise models: repeated maximal isokinetic wrist flexions (1-s) for 4 min (Wilson et al. 1988); repeated MVIC of knee extensors (3-s) once every 5-s for 5 min (Broxterman et al 2017a); sustained isometric MVIC of dorsiflexors for 4 min (Kent-Braun 1999); and maximum velocity contractions of quadriceps (every 2-s) for 4 min for young and very old female participants (Sundberg et al. 2019). Created using Biorender

Many studies have recorded peak force/power continuously in humans during various exercise regimes whilst also measuring pH_i_. This research reveals a considerable fall in pH_i_ (Table 2), yet this fact alone is insufficient to confirm that raised [H^+^]_i_ is a major factor in fatigue. One approach to address this issue is to determine whether the relationship between peak force/power and pH_i_ during fatigue is consistent across studies. Figure 3 shows data from selected studies that depict the peak force/power—pH_i_ (or -[H^+^]_i_) relationships in humans during fatiguing voluntary contractions. These fatigue models included repeated wrist flexions, repeated MVIC for knee extensors, prolonged MVIC for tibialis anterior, and repeated shortening contractions for knee extensors of young (20–25 y) and old (70–75 y) participants. No consistent peak force/power-pH_i_ relationship was seen across studies. The initial decline of peak force/power (5–10%) occurred during an intracellular alkalosis, hence, was not caused by elevated [H^+^]_i_. The variable force/power level during fatigue for a given acidosis, e.g. 90–40% of maximum at pH_i_ 6.7 (Fig. 3), can be explained if fatigue mechanisms other than lowered pH_i_ per se are involved. Indeed, in these studies and with similar exercise models, central fatigue has been shown to contribute (Broxterman et al. 2017a; Kent-Braun 1999; Hureau et al. 2022), the early loss of force/power may involve elevated P_i_ or [H_2_PO_4_^−^] (Broxterman et al. 2017a; Sundberg et al. 2019; Wilson et al. 1988), and impairment of power involves a reduced shortening velocity (Sundberg et al. 2019). Variability may also arise from different fibre-type compositions between muscles, since some muscle processes have different sensitivities to acidosis across fibre-types (Karatzaferi et al. 2017; Lynch et al. 1994; Nelson and Fitts 2014). Moreover, the relationship between force/power and pH_i_ (or [H^+^]_i_) during fatigue has routinely been described using linear associations (Adams et al. 1991; Hureau et al. 2022; Kent-Braun 1999; Messonnier et al. 2007; Sundberg et al. 2019). We emphatically argue that these associations are inappropriate given that the relationships are often non-linear (Fig. 3) and that high correlation coefficients do not strengthen or confirm cause and effect between these variables. It is now time to abort using associative correlations as a testing intervention. Instead, experiments should focus on manipulating [H^+^]/[lactate^−^] to examine their potential roles in fatigue.

### Other potential fatigue factors

It appears likely that factors/agents other than raised [H^+^]_i_ contribute to the decline of force/power with different fatigue models (Fig. 3). These fatigue factors may act directly or indirectly via interactions with raised [H^+^]_i_/[lactate^−^]_i_ to impair muscle performance. They include: (i) intracellular metabolic changes i.e., decreased concentrations of ATP (and increased [Mg^2+^]), and elevated P_i_, adenosine diphosphate, adenosine monophosphate, and inosine monophosphate (Broxterman et al. 2017a,b; Hargreaves et al. 1998; Harmer et al. 2000; Sundberg et al. 2019); (ii) reduced fuel availability, i.e. glycogen, PCr, and possibly glucose (Black et al. 2017; Kowalchuk et al. 1988a, b; Sahlin et al. 1978; Vigh-Larsen et al. 2022); (iii) run-down of trans-sarcolemmal ionic gradients for [K^+^], [Na^+^], [Ca^2+^] and [Cl^−^] (Black et al. 2017; Harmer et al. 2000; Hostrup et al. 2021; Kowalchuk et al. 1988b; Sahlin et al. 1978) and (iv) increased oxidative stress factors, i.e. the reactive oxygen species (ROS) of hydrogen peroxide and superoxide anion (Allen et al. 2008; Cooke 2007). We now introduce two well-documented fatigue factors that interact with raised [H^+^]_i_.

### Inorganic phosphate

**Fig. 4:**
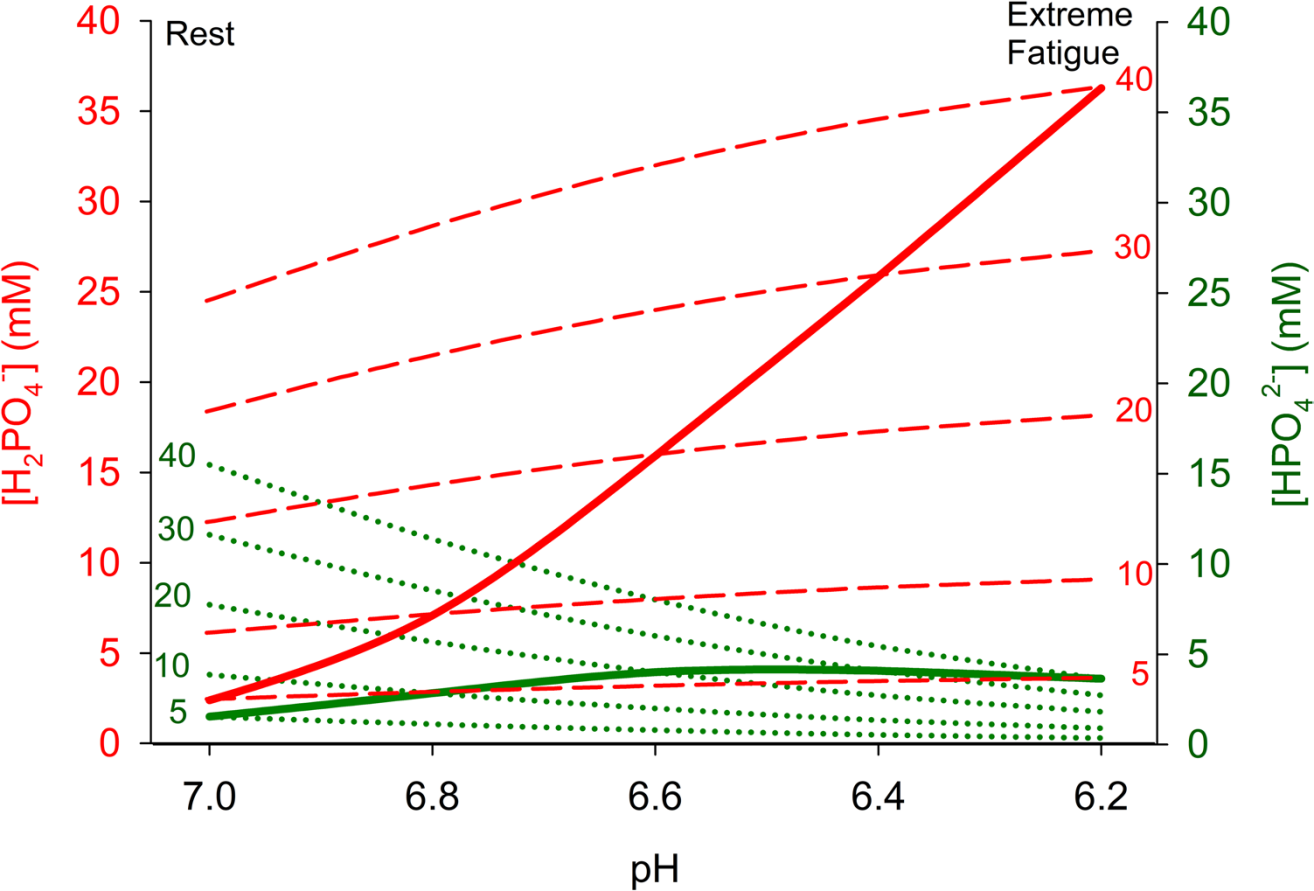
Relationships between pH and the two main ionic species of phosphate (HPO_4_^2−^, H_2_PO_4_^−^) in skeletal muscle. The isopleths show concentrations of these phosphate species at constant total [P_i_] of 5–40 mM, over the physiological pH range. The solid red line shows the increase of [H_2_PO_4_^−^] as total P_i_ increases and pH decreases with intense exercise. Increases of [HPO_4_^2−^] are minor. Phosphate species were calculated from measures of pH and total P_i_ using the equation pH = pKa + log_10_[HPO_4_^2−^]/[H_2_PO^4−^] with the pKa of 7.2. Created using Biorender

When PCr is consumed during intense exercise, the myoplasmic [P_i_] rises from 1–6 mM at rest (Kemp et al. 2007; Kushmerick et al. 1992) to 20–40 mM (Broxterman et al. 2017a,b; Hureau et al. 2022; Newham and Cady 1990; Sundberg et al. 2019). The elevated [P_i_] is proposed to cause fatigue via impaired myofilament function and/or reduced SR Ca^2+^ release (Allen et al. 2008; Dahlstedt et al. 2000; Fryer et al. 1995; Korzeniewski 2019). Notably, P_i_ exists as four molecular species (PO_4_^3−^, HPO_4_^2−^, H_2_PO_4_^−^, H_3_PO_4_) with their concentrations determined by their acid dissociation constant, pH and temperature (Kushmerick 1997)-HPO_4_^2−^ and H_2_PO_4_^−^ being the main species. With severe acidosis, i.e., pH 6.6–6.2, the [H_2_PO_4_^−^] is predicted to reach 20–40 mM with [HPO_4_^2−^] being largely unchanged at ~5 mM (Fig. 4). Indeed, H_2_PO_4_^−^ is the most abundant P_i_ species in fatigued muscle where it can reach 10–25 mM (Broxterman et al. 2017a,b; Hureau et al. 2022; Kent-Braun 1999; Newham and Cady 1990; Sundberg et al. 2019; Weiner et al. 1990). Moreover, Nosek et al. (1987) found in skinned fibres that raised [H_2_PO_4_^−^] (or total [P_i_]) correlated better than decreased pH to the decline of maximum Ca^2+^-activated force, i.e. the force when troponin-C is saturated with Ca^2+^. Hence, they proposed [H_2_PO_4_^−^] to be a major fatigue culprit. Interestingly, raised [P_i_] reduces maximum force at pH 7.0 (Debold et al. 2006; Fryer et al. 1995; Karatzaferi et al. 2003), where the main species is HPO_4_^2−^ (Fig. 4). Hence it remains to be determined whether protonation to H_2_PO_4_^−^ is a necessity to reduce force during fatigue.

**Fig. 5:**
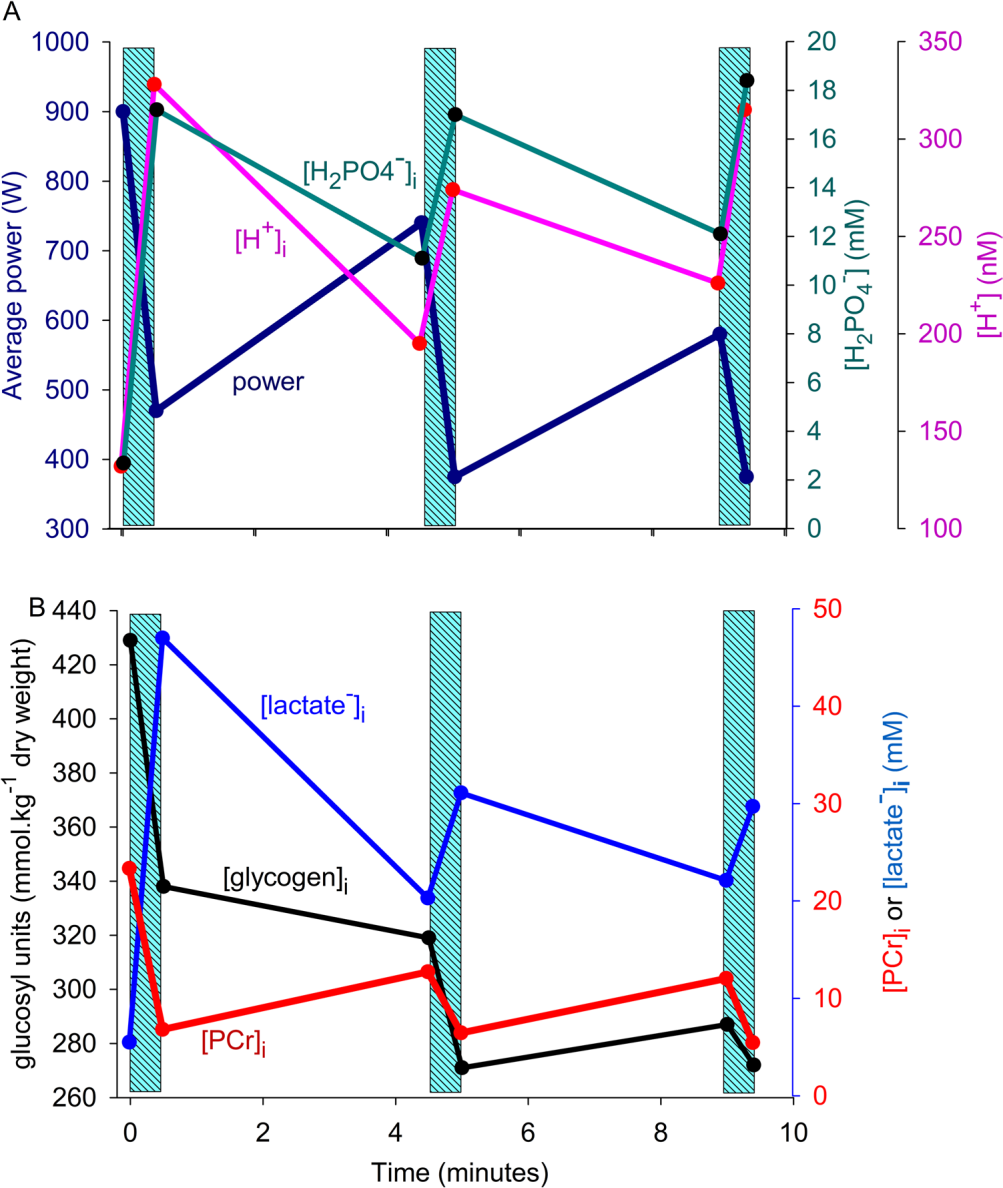
Effects of three 30-s bouts of very high-intensity cycling exercise by humans, interspersed with 4-min rest intervals, on power and muscle metabolites implicated in fatigue processes. **A** Significant recovery of average power, [H^+^]_i_ and [H_2_PO_4_^−^]_i_ occurred between exercise bouts giving an appearance of cause-effect. Because of this acidosis most of the total [P_i_]_i_ exists as [H_2_PO_4_^−^]_i_. Associations between these three variables diminish during bouts 2 and 3. **B** PCr hydrolysis and glycogenolysis/glycolysis are required to meet the ATP demand during the first bout, yet power at the start of bout 2 is high while glycogen and PCr remain low, and [lactate^−^]_i_ is elevated. Data from McCartney et al. 1986 and Kowalchuk et al. 1988a, b. Created using Biorender

The changes in metabolite concentrations, as related to power, during the rest intervals of repeated bouts of highintensity exercise provide additional insight into the relationship between variables (Fig. 5). Calculated [H_2_PO_4_^−^]_i_ during repeated all-out 30-s cycling bursts increased to 18 mM at a time when average power had fallen to ~40% of initial. The decline of power from the first to the end of the third exercise bout relates equally well to increased [H_2_PO_4_^−^]_i_ and [H^+^]_i_. When one ignores the recovery periods it appears that [H_2_PO_4_^−^]_i_ and [H^+^]_i_ reached a steady level from the end of bout 1. However, the magnitude of the power increases in rest intervals 2 and 3 are not proportional to the increase in P_i_ species compared to the increases that occurred in the first bout, and the loss of power in bouts 2 and 3 are also disproportionate to the increase in P_i_ species. While one may be unable to discriminate between the [H^+^]_i_ and [H_2_PO_4_^−^]_i_ effects on power, it appears that the size of the effect is determined by other factors.

### Ionic changes

K^+^-disturbances always occur concomitantly with acidosis during intense exercise; muscle interstitial [K^+^]_o_ increases from 4 to 7–15 mM whilst [K^+^]_i_ decreases from 160 to 100–130 mM (Gunnarsson et al. 2013; Hostrup et al. 2021; Kowalchuk et al. 1988b; Renaud et al. 2023). In addition, lowered [K^+^]_i_ contributes significantly to the rise of [H^+^]_i_ during exercise (Heigenhauser and Lindinger 1988; Kowalchuk et al. 1988a; Stewart 1983). Acidosis also promotes K^+^ efflux through ATP-sensitive potassium (K_ATP_) channels during exercise (Hostrup et al. 2021; Renaud et al. 2023; Street et al. 2005). Reduced K^+^-gradients depolarise the sarcolemma which then depress action potential amplitude to impair Ca^2+^ release from the SR (Cairns et al. 2022; Wang et al. 2022) and renders some fibres inexcitable (Cairns et al. 2022; Juel 1988b). Certainly, large K^+^-disturbances per se reduce muscle force (Bandschapp et al. 2012; Cairns et al. 2015, 2022; de Paoli et al. 2010; Renaud et al. 2023) whereas smaller K^+^-changes evoke potentiation (Cairns et al. 2022; Olesen et al. 2021; Renaud et al. 2023). Intimately linked to K^+^-efflux is the Na^+^ influx which occurs during each action potential, and which eventually reduces the trans-sarcolemmal Na^+^-gradient (Lindinger et al. 2024; Sahlin et al. 1978). The resulting K^+^-Na^+^ interaction further impairs action potentials beyond the effects of K^+^ alone to exacerbate force depression (Cairns et al. 2022; Overgaard et al. 1999).

### Physiological processes

Detrimental changes occur to muscle and CNS processes during intense exercise. This comprises impairment of compound muscle action potentials (M-wave) (Black et al. 2017), maximal Na^+^-K^+^-ATPase activity (Hostrup et al. 2014; Vigh-Larsen et al. 2025), SR Ca^2+^ release (RyR1 channel) (Hostrup et al. 2014; Olsson et al. 2020), SR Ca^2+^ uptake (Ca^2+^-ATPase, or SERCA activity) (Cairns et al. 2017; Hargreaves et al. 1998; Hostrup et al. 2014), and voluntary activation (Hureau et al. 2022; Kent-Braun 1999). These processes may potentially become sensitive to H^+^/lactate^−^. Moreover, plasma catecholamines (adrenaline, noradrenaline) and muscle sympathetic activity become elevated with intense exercise (Hargreaves et al. 1998; Harmer et al. 2000; MacLean et al. 2000; Nielsen et al. 1999). These hormones modify glycogenolysis/glycolysis, ionic balance, action potentials, and Ca^2+^ handling (de Paoli et al. 2007; Hansen et al. 2005; Hostrup et al. 2014; Pedersen et al. 2003) to potentially modify H^+^-effects.

## Interventions to test effects of lactate^−^ and/or acidosis on performance

Experiments to assess the effects of raised [H^+^]/[lactate^−^] are best performed in the normal in vivo physiological range for these two ions (Tables 1 and 2) and must impair muscle/exercise performance to be regarded as genuine factors in fatigue. Common measures used in testing include force (peak MVIC force, peak tetanic and twitch force, and rates of force rise or relaxation), shortening velocity (maximal shortening speed, i.e. V_max_, velocity in slack test, or with investigation of the force–velocity relationship), the resulting power (power = force × velocity), and exercise performance time (Cairns 2013; Knicker et al. 2011). Notably, the functionally important peak power measure is understudied in human and animal exercise science research. Experiments have often utilised non-fatigued/resting humans or muscle preparations to directly test effects of H^+^/lactate^−^. One must be aware of limitations when attempting to translate such results from non-fatigued muscle to what occurs in contacting human muscle in vivo (e.g. Kristensen et al. 2005; Watanabe and Wada 2020) since some conditions change, e.g. muscle processes, enzyme activities, and muscle environments. Experiments entailing resting conditions have been done on muscle in situ or in vitro or by mimicking fatigue milieu with skinned fibres or isolated muscle proteins. Other studies have involved fatigue during whole-body or single-muscle exercise in humans, or with electrical stimulation of muscles in situ or in vitro.

**Fig. 6:**
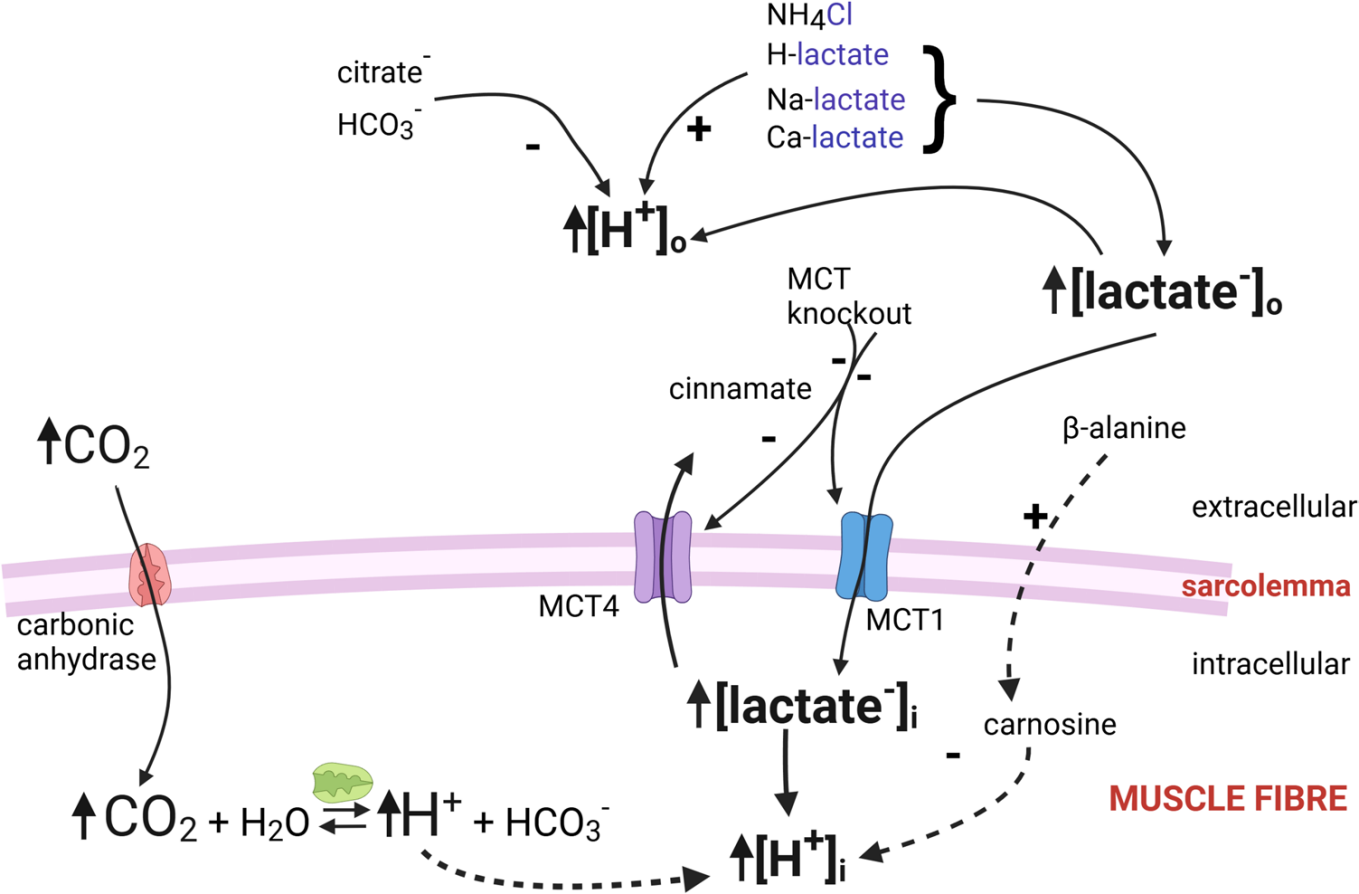
Interventions commonly used to test effects of raised [lactate^−^] or [H^+^] on muscle/ exercise performance. These include a metabolic acidosis induced with exogenous application of ammonium chloride, lactic acid, Na-lactate, Ca-lactate, or lowered [HCO_3_^−^]; a respiratory-acidosis induced with raised CO_2_. Tests on skinned fibres involve direct addition of H^+^ (as EGTA). An exercise-induced acidosis may be countered with an alkalosis with Na-HCO_3_, Na-citrate (extracellular H^+^-buffers), or β-alanine—a precursor for carnosine (intracellular H^+^-buffer). Sarcolemmal monocarboxylate lactate^−^ transporter proteins (MCT1, MCT4) can be blocked with cinnamate or genetically modified. Created using Biorender

Figure 6 shows interventions routinely used to test for the role of H^+^/lactate^−^ in fatigue. A pre-exercise induced acidosis is postulated to accelerate fatigue by exacerbating the exercise-induced acidosis and/or through interactions with other fatigue factors. Such treatments have included ingestion, infusion, or superfusion with sodium-lactate (Na-lactate), calcium-lactate (Ca-lactate), or lactic acid (H-lactate), all of which increase [lactate^−^]_o_ and lower pH_i_ (Overgaard et al. 2010) following lactate^−^ entry into muscle fibres via MCT. However, the resulting changes of [lactate^−^]_i_ and hence pH_i_ are variable (Gladden and Yates 1983). Raised extracellular lactic acid also directly lowers pH_o_ (Overgaard et al. 2010). Respiratory-acidosis can be induced with raised CO_2_ in extracellular fluids (in HCO_3_^−^ buffered saline solutions), with CO_2_ then diffusing into the myoplasm, where via carbonic anhydrase and reactions with water, it elevates [H^+^]_i_. The pH_i_ falls progressively with increasing extracellular CO_2_ (Adams et al. 1991) which is usually from 0 to 5–10% CO_2_ for humans (Mador et al. 1997; McCartney et al. 1983; Vianna et al. 1990) and 5 to 20–70% CO_2_ for animal muscles (Adams et al. 1991; Harkema et al. 1997; Meyer et al. 1991; Westerblad et al. 1997). Note that the latter acidosis involves experimental rather than physiological CO_2_ levels. A metabolic-acidosis can be induced with ammonium chloride (NH_4_Cl) (e.g. 3 g/kg body weight) which exacerbates development of intracellular acidosis by 0.1–0.2 pH units during intense contractile activity (Churchward-Venne et al. 2010; Hollidge-Horvat et al. 1999; Hultman et al. 1985). NH_4_Cl is largely without effect on pH_i_ at rest (Hollidge-Horvat et al. 1999; Hultman et al. 1985; Kowalchuk et al. 1984) and does not alter the maximal exercise-induced intracellular acidosis (Hood et al. 1988). Metabolic acidosis can also be induced with lowered [HCO_3_^−^]_o_ (e.g., from 24 to 13 mM) (Kowalchuk et al. 1988a; Spriet et al. 1985), or L-arginine-hydrochloride or hydrochloric acid added to the superfusate around animal muscle in situ or in vitro (e.g., Hirche et al. 1975; Steinhagen et al. 1976). When testing with skinned fibres or isolated myosin proteins, H^+^/lactate^−^ are added directly to the bathing milieu (cytoplasmic environment) under highly buffered conditions so that precise concentrations are known (Debold et al. 2008; Karatzaferi et al. 2008; Lamb and Stephenson 1994). Commonly used chemical skinning, where the surface sarcolemma is permeabilised, allows direct evaluation of myofilament function, whereas mechanical skinning (peeling) with sealed T-tubules, also allows assessment of excitation–contraction coupling and T-system membrane excitability (Lamb and Stephenson 2018). Finally, it is possible to manipulate H^+^-regulation processes (Fig. 6). For example, with added NaHCO_3_ as an extracellular H^+^-buffer (De Oliveira et al. 2022; Grgic et al. 2020), with pharmacological or genetic manipulation of sarcolemmal MCT isoforms (Bisetto et al. 2019; Kitaoka et al. 2022) or carbonic anhydrase isoforms (Feng and Jin 2016; Liu et al. 2007).

## Effects of lactate^−^ on muscle and exercise performance

Lactate^−^ is not a waste product of metabolism but rather it has important beneficial roles as an oxidizable energy substrate and gluconeogenic precursor via the lactate shuttle (Brooks 2018), as a signaling molecule for muscle adaptations (Brooks et al. 2023; Ferguson et al. 2018), and as a substrate for mitochondrial respiration (Brooks et al. 2022). We now evaluate studies that have tested for a specific role of lactate^−^ in fatigue, rather than its indirect effects via a lactate-induced acidosis.

### Extracellular lactate^−^ and fatigue

#### Human studies

Many studies indicate that raised [lactate^−^]_o_ is not detrimental for force/power generation during exercise. First, there is no positive correlation between increased plasma [lactate^−^]_o_ (over 1–20 mM) and the decline of peak power during a supramaximal trial with elite cyclists (Mildenhall et al. 2023). Second, plasma [lactate^−^]_o_ can become further elevated post exercise (Costill et al. 1983; Harmer et al. 2000; Kowalchuk et al. 1988a), at a time when force/power is recovering. Furthermore, the addition of [lactate^−^]_o_ can have positive ergogenic effects. Pre-exercise Na-lactate or Ca-lactate ingestion can prolong the time to exhaustion with intense treadmill running (van Montfoort et al. 2004) or cycling (Morris et al. 2011), although this effect is not always seen (de Salles Painelli et al. 2014). Lactate^−^ ingestion via sports drinks also permitted a more prolonged intense cycling burst after 90 min of submaximal exercise (Azevedo et al. 2007). Notably, these positive ergogenic effects were coupled to a raised plasma [HCO_3_^−^] (Morris et al. 2011; van Montfoort et al. 2004) which presumably results from transport of lactate^−^ into cells then its oxidation (Gladden and Yates 1983), thus necessitating an increase in balancing negative charge in plasma. Thus lactate^−^ supplementation may enhance performance through an extracellular alkalising effect that protects against raised [H^+^]_i_. On-the-other-hand, when [lactate^−^]_o_ was elevated to ~10–12 mM, due to altered fractions of inspired oxygen (Hogan and Welch 1984) or prior exercise by another muscle group, e.g. arm exercise before leg work (Bangsbo et al. 1996; Bogdanis et al. 1994; Nordsborg et al. 2003; Yates et al. 1983) then there was little effect on peak MVIC force (Jacobs et al. 1993; Yates et al. 1983) or peak power (Bogdanis et al. 1994), yet time to exhaustion was abbreviated (Bangsbo et al. 1996; Nordsborg et al. 2003; Yates et al. 1983).

#### Animal muscle studies

Exposing isolated non-fatigued animal muscle preparations to raised [lactate^−^]_o_ has yielded equivocal results. Raised [lactic acid] or [Na-lactate] (10–24 mM) is without effect on peak tetanic force in isolated slow-twitch soleus and fast-twitch extensor digitorum longus (EDL) muscles of mice (25–35 °C) (Phillips et al. 1993; Spangenburg et al. 1998; Zhang et al. 2006). Other studies report that Na-lactate (10–50 mM) can lower tetanic force by 15–20% in soleus, EDL and diaphragm muscles of rodents (25–37 °C) (Coast et al. 1995; Erdoğan et al. 2002; Kristensen et al. 2005; Spangenburg et al. 1998). However, there needs to be caution when using high [lactate^−^]_o_ due to potential deleterious effects of increased osmolarity (Allen et al. 2008; Chase and Kushmerick 1988). In further contrast, raised [lactic acid] augmented the peak force of dynamic contractions (8%) in rat soleus (30 °C), whilst reducing V_max_ (9%) with maximum power being unchanged (Overgaard et al. 2010). These variable effects of raised [lactate^−^]_o_ may have arisen due to different [lactate^−^]_i_ and pH_i_ levels with these interventions (Chin et al. 1997; Gladden and Yates 1983).

**Fig. 7:**
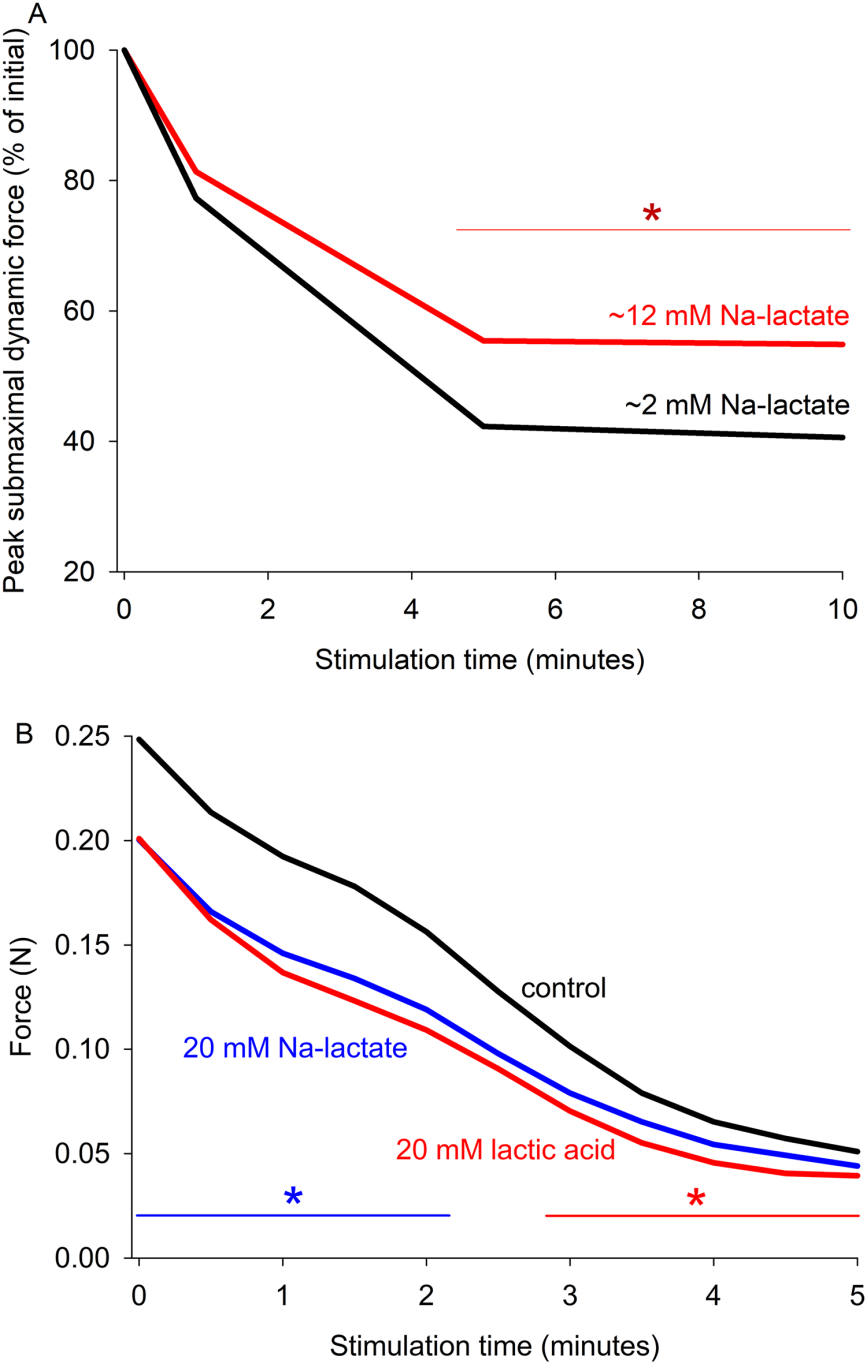
Influence of lactate^−^ or lactic acid on the fatigue profile of **A** rat gastrocnemius muscle stimulated in situ (Karelis et al. 2004) and **B** rat soleus muscles stimulated in vitro (Kristensen et al. 2005) In A, continuous infusion of Na-lactate (~ 12 mM in plasma) from time 0 stimulation, fatigue protocol: stretch then nerve stimulation (50 Hz for 200 ms while shortening), every 2.7 s for 60 min. Data beyond 10 min are not shown because no further changes occurred. Peak submaximal dynamic force was measured, 36 °C. In B, isolated muscles were bathed in test solutions (20 mM Na-lactate, 20 mM lactic acid) for 15 min, followed by repeated isometric contractions (33 Hz for 1 s), every 3-s for 5 min total duration. Peak submaximal isometric force was measured, 30 °C. Created using Biorender

Several studies have examined the effects of raised [lactate^−^]_o_ on fatigue kinetics. Rapid infusion of Na-lactate (to 14 mM) into blood perfusing dog gastrocnemius muscle in situ resulted in a faster decline of force during continuous twitch stimulation, and subsequent removal of [lactate^−^]_o_ elicited force recovery (Hogan et al. 1995). This may be interpreted as extracellular lactate^−^ being detrimental. In opposition, a thorough study on rat gastrocnemius muscle in situ showed that infusion of Na-lactate (to 14 mM) protected against the decline of the M-wave and slowed the decline of tetanic force during repeated shortening contractions by 15–20% (Fig. 7A) (Karelis et al. 2004). Similarly, Na-lactate (20 mM) slowed fatigue at 8 mM [K^+^]_o_ during a prolonged tetanus in rat soleus muscle in vitro (Clausen and Nielsen 2007). In line with these latter findings, exposure to 5–20 mM Na-lactate or lactic acid restored force in K^+^-depressed muscles (de Paoli et al. 2007, 2010; Hansen et al. 2005; Kristensen et al. 2005; Nielsen et al. 2001); an effect attributed to a reduced sarcolemmal chloride (ClC-1) channel conductance (Bandschapp et al. 2012; de Paoli et al. 2010; Nielsen et al. 2001). Taken together, these findings show that lactate^−^ can be protective for both K^+^-depressed and fatiguing muscle. However, 10 mM lactic acid did not modify the fatigue profile during repeated tetani in isolated mouse muscles at 35 °C (Zhang et al. 2006). Also, pre-incubation with either 20 mM of Na-lactate, lactic acid or a lactate^−^/lactic acid mix (all of which also lower pH_i_) exerted small detrimental effects on fatigue kinetics during repeated isometric tetani in isolated rat soleus muscles, 30 °C (Fig. 7B). Consistent with the latter findings, prior exercise by one muscle group to elevate plasma [lactate^−^]_o_ reduced the total work done (Jacobs et al. 1993). Interpretation of these studies is complex because of the different [lactate^−^]_o_ used, variable elevations of [lactate^−^]_i_ and [H^+^]_i_, an extracellular alkalinising effect, enhanced muscle blood flow (Gladden and Yates 1983), different ratings of perceived exertion (RPE) (Hogan and Welch 1984), and changes to other cellular processes including greater sarcolemmal K^+^ release (Nordsborg et al. 2003). More research is needed with pH_i_ measurements to clarify and explain these diverse findings.

### Intracellular lactate^−^ and fatigue

Recovery studies show that when allowing a 2–3 min period after a prolonged MVIC or all-out sprinting, the [lactate^−^]_i_ in human quadriceps muscle remains elevated (23–33 mM), yet the peak force/power had almost fully recovered to pre-exercise levels (Bogdanis et al. 1995; Sahlin and Ren 1989). Thus, high [lactate^−^]_i_ does not cause fatigue. A more direct approach involves using skinned animal muscle fibres to test the effects of raised [L( +)-lactate^−^]_i_ (at normal pH). Exposure to 15–30 mM [lactate^−^]_i_ induced a small but significant decline, ~ 4% (range 2–6%), of maximum Ca^2+^-activated force in skinned rat or rabbit muscle fibres (22–24 °C) (Andrews et al. 1996; Dutka and Lamb 2000; Posterino and Fryer 2000; Posterino et al. 2001). When using T-system action potentials and hence normal voltage activation to trigger Ca^2+^ release from the SR, application of 30 mM lactate^−^ reduced maximal force to 91% of control (Dutka and Lamb 2000). Hence raised [lactate^−^]_i_, as described in Table 1, causes at the most a minor reduction of peak force.

#### Summary

Raised intracellular (or extracellular) [lactate^−^] has little direct effect on peak force. At the most raised [lactate^−^]_i_ results in a 2–9% lower force during fatigue.

## Effects of intracellular acidosis on muscle and exercise performance

### Non-fatigued conditions

#### Human studies

**Fig. 8:**
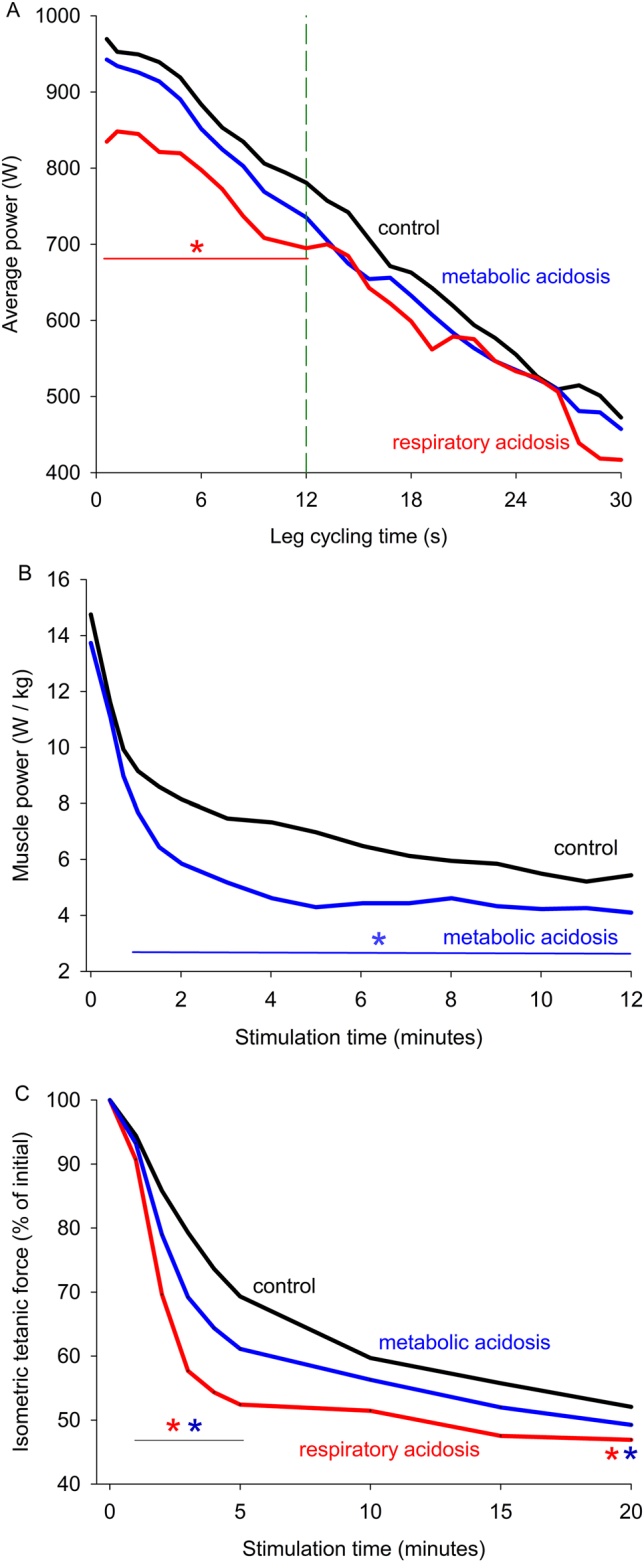
Influence of an induced acidosis on the fatigue profile during intense human exercise or with animal muscles stimulated in situ. **A** Human average power measured during all-out isokinetic cycling for 30-s in males during respiratory-acidosis (5% CO_2_), metabolic-acidosis (NH_4_Cl 0.3 g/kg body wt) or placebo control (CaCO_3_). Vertical dashed green line indicates the time point at which there is no effect of induced acidosis on power (McCartney et al. 1983). **B** Muscle power measured in dog gastrocnemius, fatigue protocol: nerve stimulation (100 Hz for 200 ms with shortening contractions), then 500 ms rest, repeated for 12 min, 37 °C. Metabolic-acidosis induced with infusion of L-arginine hydrochloride (Steinhagen et al. 1976). **C** Peak isometric force measured in rat gastrocnemius-plantaris-soleus muscle group, fatigue protocol: nerve stimulation (100 Hz) once every 2-s for 20 min, 37 °C. Metabolic acidosis induced by decreasing [HCO_3_^−^] from 23.6 to 12.9 mM. Respiratory acidosis induced by increasing arterial PCO_2_ from 37.7 to 63.0 mmHg (Spriet et al. 1985). * indicates time points significantly lower with acidosis than control, for all panels. Created using Biorender

A respiratory-acidosis (with inspired 5% CO_2_) evoked a 9% lower peak power during all-out isokinetic cycling (Fig. 8A). Similar exposure to 8–9% CO_2_ reduced the peak isometric tetanic force by 13–20% in adductor pollicus and quadriceps muscles in vivo although there was no effect on the diaphragm (Mador et al. 1997; Vianna et al. 1990). In contrast, when a 30% CO_2_-induced acidosis (pH_i_ ~ 6.7) was imposed on isolated human intercostal fibres (37 °C), there was no effect on the peak force of maximal or submaximal contractions, yet relaxation was prolonged (Olsson et al. 2020). Likewise, in chemically skinned human vastus lateralis fibres, a lower pH of 6.6 had no significant effect on maximum Ca^2+^-activated force (22 °C) (Lynch et al. 1994). When exploiting a prior exercise-induced acidosis using a sustained MVIC or repeated contractions (adductor pollicis, wrist flexors, quadriceps), then with a subsequent 2–5 min recovery period the pH_i_ remained low at 6.7–6.5 yet there was almost full recovery of peak MVIC force (i.e. to 89–100% initial) (Hureau et al. 2022; Miller et al. 1987; Sahlin and Ren 1985; Wilson et al. 1988) and peak power (Bogdanis et al. 1995). Also, electrical stimulation studies have shown that peak muscle force can be maintained (>90% initial) despite an acidosis to pH_i_ 6.8–6.65 (Chasiotis et al. 1987; Spriet et al. 1987a,b). Together, these data indicate that a reduced pH_i_ to ~6.7 per se has little depressive effect (0–13% decline) on peak force/power in human muscle.

#### Animal muscle studies

When an acidosis is induced in animal muscles in situ (30–37 °C) with raised CO_2_, L-arginine-hydrochloride, or lowered [HCO_3_^−^], there was little effect on peak (initial) muscle force/power for dog gastrocnemius (Fig. 8B), rat hindlimb (Fig. 8C), and cat biceps and soleus muscles (Adams et al. 1991; Harkema et al. 1997; Meyer et al. 1991). When using 30–70% CO_2_ with these cat muscles the pH_i_ fell to ~6.5, peak tetanic force fell by 6% (0–16%) and peak twitch force fell by 32%, whilst relaxation was slowed. When a myoplasmic acidosis is induced at low temperatures (10–20 °C), i.e., to pH_i_ 6.8–6.6 in isolated intact rodent muscles/fibres (Sahlin et al 1983; Wiseman et al. 1996; Westerblad et al. 1997) or to pH 6.2 in skinned fibres (Knuth et al. 2006; Pate et al. 1995), a marked decline of maximum isometric force occurs, i.e., ~32% (range 20–53%). At physiological muscle temperatures of 28–40 °C (Bruton et al. 1998; Krustrup et al. 2006; Spriet et al. 1989), the decline of peak isometric force and V_max_ were both attenuated. This observation prompted the question of whether an intracellular acidosis per se causes much fatigue (Allen et al. 2008; Lamb and Stephenson 2006; Westerblad 2016). Wiseman et al. (1996), using 25% CO_2_, carefully quantified a breakpoint showing greater H^+^-induced force loss at temperatures below 21 °C in mouse EDL muscle. Only small differences appeared for H^+^-effects at temperatures between 22 and 32 °C (Westerblad et al. 1997). Based on these studies, experiments at <20 °C should be avoided when quantifying pH effects on muscle function in order to have meaning for humans in vivo. In summary, intracellular acidosis in situ exerts minor effects per se on the maximal isometric force.

**Fig. 9:**
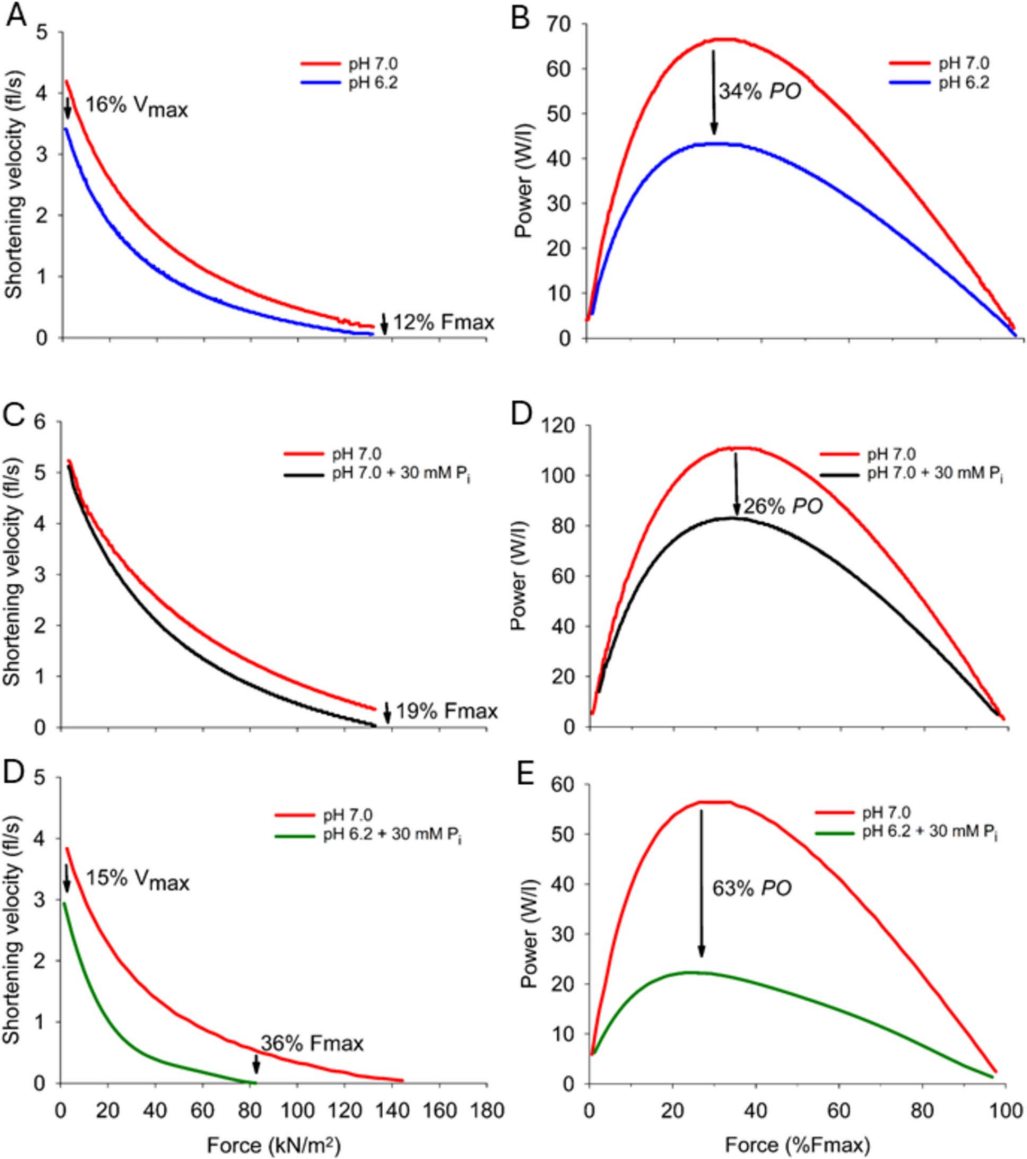
Influence of lowered pH and raised inorganic phosphate (P_i_) individually and combined, on the force–velocity relationship and maximum power in skinned rat muscle fibres. **A**, **B**: Effects of lowered pH (7.0–6.2) per se to reduce maximal velocity (V_max_), maximal isometric force (F_max_), and peak power output (PO). **C**, **D**: Effects of raised [P_i_] (5–30 mM) per se to reduce F_max_, without effect on V_max_, and reduce PO. **E**, **F** Combined effects of pH 6.2 + 30 mM P_i_, to reduce V_max_, F_max_, and PO. Data are from slow-twitch type I fibres, 30 °C (Debold et al. 2016). Qualitatively similar findings occurred in fast-twitch type II fibres. Created using Biorender

Several studies have tested the effects of induced-acidosis on non-fatigued intact muscle fibres in vitro at physiological temperatures of 28–37 °C. With a CO_2_-induced acidosis (pH_i_ 6.8–6.6) the peak isometric tetanic force was reduced by ~7% (range 2–15%) (Bruton et al. 1998; Overgaard et al. 2010; Westerblad et al 1997). Similarly, with severe acidosis to pH 6.2 in skinned fast-twitch and slow-twitch fibres, the maximum Ca^2+^-activated force fell by ~12% (range 3–22%, Fig. 9A) (Karatzaferi et al. 2008; Knuth et al. 2006; Lamb and Stephenson 1994; Nelson and Fitts 2014; Pate et al. 1995). All these acidosis effects on maximum isometric force were small but likely to be functionally important. In addition, acidosis to pH_i_ 6.7–6.2 in intact or skinned fibres lowered V_max_ by ~5% (+6 to −16%, Fig. 9B) (Karatzaferi et al. 2008; Knuth et al. 2006; Nelson and Fitts 2014; Overgaard et al. 2010; Westerblad et al. 1997). These effects of raised [H^+^]_i_ per se on force and velocity, along with a greater curvature of the force–velocity relationship, reduces peak power by ~22% (range 7–39%) (Fig. 9B) (Karatzaferi et al. 2008; Knuth et al. 2006; Nelson and Fitts 2014; Overgaard et al. 2010). Further research is needed to investigate effects of acidosis on maximum force, V_max_ and peak power, across the physiological range of pH_i_ from 6.7 to 6.2.

### Fatigue conditions

#### Human studies

Respiratory-acidosis induced by inspiring 5% CO_2_ diminished the total work done to 91% of control during 30-s of all-out isokinetic cycling (Fig. 8A). However, respiratory acidosis, induced with CO_2_ rebreathing, attenuated the loss of contraction speed (by 5–10% control) during repeated concentric handgrip contractions (Hilbert et al. 2012). Furthermore, a 30% CO_2_-induced acidosis in human intercostal fibres in vitro did not significantly alter the fatigue profile during repeated tetani, although fatigue-sensitive fibres appeared to fatigue more rapidly (Olsson et al. (2020). This observation warrants further investigation. Strong evidence for the role of acidosis is provided in studies that used pre-exercise ingestion of NH_4_Cl. The findings included: impaired performance in 4-km cycling time trials with mean power output being lower and performance time prolonged (~11 s) (Correia-Oliveira et al. 2017); ~10% lower mean power output over the final 2-min of a 6-min rowing trial in elite oarsmen (Brien and McKenzie 1989); reduced power at exhaustion and reduced time to task failure during progressive incremental cycling (Kowalchuk et al. 1984) and continuous intense cycling (George and MacLean 1988; Jones et al. 1977; Robergs et al. 2005; Sutton et al. 1981); and reduced total work achieved during repeated leg extensions (Jacobs et al. 1993). Importantly, NH_4_Cl ingestion exacerbated the loss of peak force from 55 to 45% initial and caused pH_i_ to fall from 6.70 to 6.54 during continuous tetanic stimulation of quadriceps (Hultman et al. 1985). The consistent findings of impaired performance with NH_4_Cl likely involve a 0.1–0.2 pH unit greater acidosis during intense exercise (Churchward-Venne et al. 2010; Hollidge-Horvat et al. 1999; Hultman et al. 1985).

#### Animal studies

An induced acidosis in situ with L-arginine-hydrochloride or hydrochloric acid for dog gastrocnemius (Fig. 8B, Hirche et al. 1975), with a metabolic- or respiratory-acidosis (lowered [HCO_3_^−^]) in rat hindlimb muscles (Fig. 8C), and with 30% CO_2_ in mouse soleus (Feng and Jin 2016), all hastened the decline of force/power early during repeated tetanic stimulation (by 5–30% initial). Hence three different approaches to induce acidosis yield support for the hypothesis that raised [H^+^]_i_ contributes to force/power loss during this fatigue. With isolated mouse soleus muscles (30 °C) pre-exposure to 20 mM Na-lactate for 15 min, which also lowered pH_i_, exacerbated the early force loss during repeated tetanic stimulation, whereas 20 mM lactic acid (lowers both pH_o_ and pH_i_) exacerbated the late force loss (Fig. 7B). These combined findings provide strong support for the role of acidosis in fatigue of animal muscles. However, 10 mM lactic acid did not alter the fatigue profile during repeated tetani in isolated mouse soleus or EDL (Zhang et al. 2006). Similarly, a 30% CO_2_ acidosis failed to significantly alter the number of tetani required to depress force to 40% initial in flexor digitorum brevis (FDB) fibres, albeit with a tendency for greater fatigue resistance in some fibres (Bruton et al. 1998). The disparity between these findings needs resolution.

#### Summary

Intracellular acidosis to ~pH_i_ 6.7 in human muscle, during recovery from exercise or with a CO_2_-induced acidosis, resulted in a 0–14% decline of peak force/power. With non-fatigued animal muscles in situ or in vitro (at physiological temperatures), a CO_2_-induced acidosis (pH_i_ 6.7–6.5) lowered peak tetanic force (~6%) and acidosis to pH_i_ 6.2 in skinned fibres reduced maximum force by ~12% (range 3–22%). Hence an acidosis to pH_i_ 6.7–6.2 per se causes a small decline of muscle performance. An NH_4_Cl-induced acidosis impairs human muscle/exercise performance during high-intensity exercise lasting 30 s to 15 min. Also, metabolic- or respiratory acidosis accelerated fatigue of animal muscles in situ during early stages of stimulation which supports a contribution from acidosis to fatigue (at least 5–30% initial).

## Interactive effects of intracellular acidosis with other fatigue factors on muscle performance

### Acidosis and potassium

**Fig. 10:**
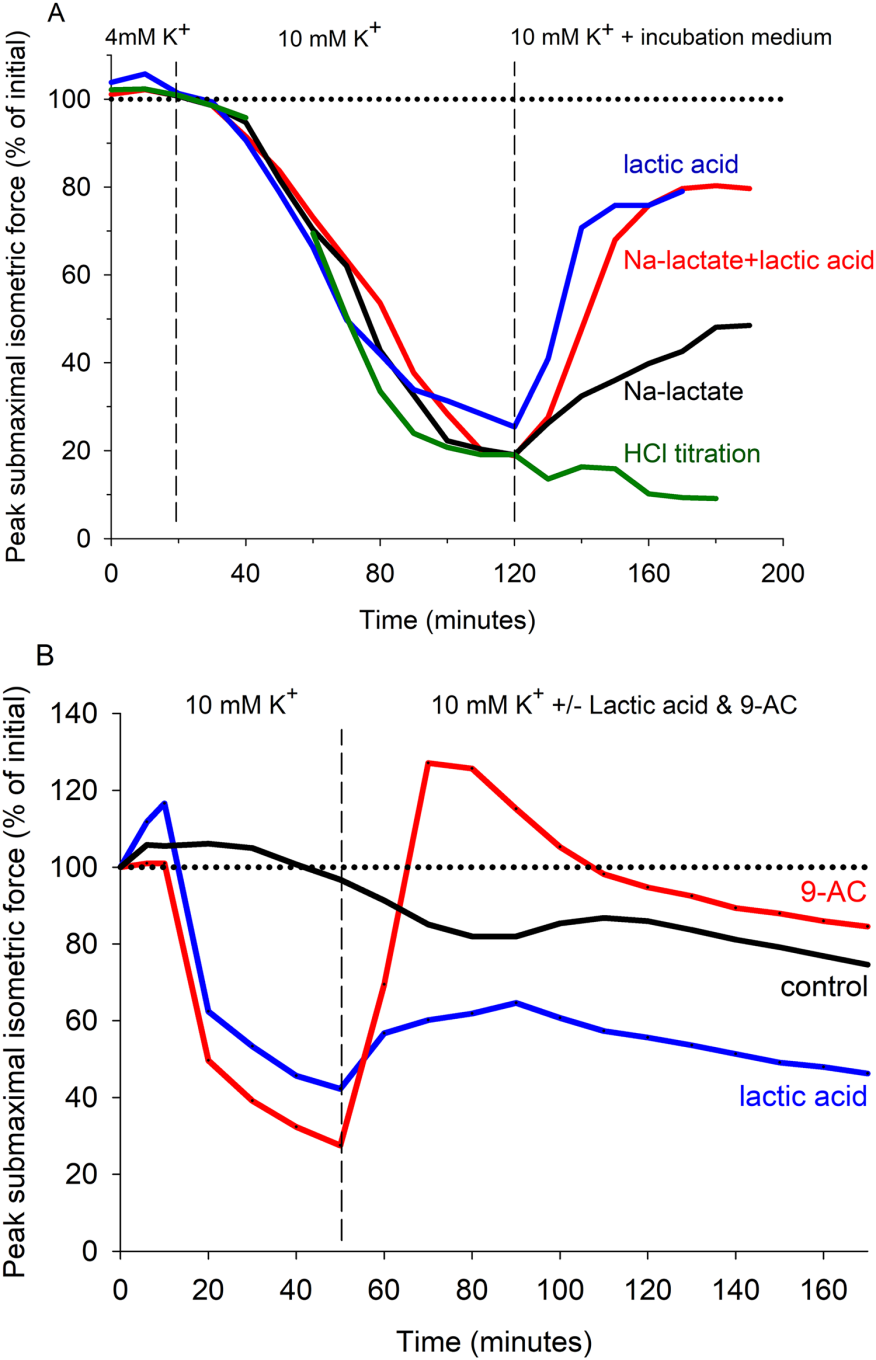
Force restoring effects of an induced acidosis on K^+^-depressed force in isolated resting muscle from **A** rat (Kristensen et al. 2005) or **B** humans (Bandschapp et al. 2012). In **A**, isolated rat soleus muscles were bathed first at 4 mM K^+^ then [K^+^] was increased to 10 mM. Isometric tetani (30 Hz for 1.5 s) were evoked every 10 min until a steady force was achieved at 100 min exposure. Muscles were then exposed to 20 mM (lactic acid, Na-lactate, Na-lactate/lactic acid mix, or HCl) at 30 °C. In **B**, isolated human vastus lateralis fibre bundles were bathed first at 4.6 mM K^+^ then [K^+^] was increased to 10 mM for 50 min. Isometric tetani (33 Hz) were evoked once every 10 s. Preparations were then exposed to 20 mM lactic acid, or ClC-1 channel blocker, 9-AC. Time control is shown. Created using Biorender

Nielsen et al. (2001) were the first to test for a combined effect of lactic acid and raised [K^+^]_o_ on contraction using isolated rat soleus muscles. They found that when non-fatigued muscles were incubated at 11 mM [K^+^]_o_ (mimicking interstitial [K^+^] during intense exercise) the isometric tetanic force was severely depressed. Then an intracellular acidosis was induced, with 20 mM lactic acid (or propionic acid) or 23% CO_2_, which resulted in a large recovery of both M-wave area and force. These findings for intact slow-twitch rodent muscle have been replicated (Fig. 10A; de Paoli et al. 2007; Overgaard et al. 2010; Pedersen et al. 2005) and also shown for intact fast-twitch muscle (Hansen et al. 2005), skinned fast-twitch fibres (Pedersen et al. 2004), and human muscle fibre bundles (Fig. 10B; Bandschapp et al. 2012; Lehmann-Horn et al. 1987). The depression of power for dynamic contractions at raised [K^+^]_o_ is ameliorated with acidosis; shortening velocity was unchanged which implies that power restoration involved an increased force (Overgaard et al. 2010). Protective effects on force were quantitatively similar over a range of pH_i_ from 6.8 to 6.4 (Hansen et al. 2005). A noteworthy point is that induced acidosis does not increase force when K^+^ disturbances are moderate, e.g. 7 mM [K^+^]_o_ (Olesen et al. 2021) or extremely large, e.g. >14–16 mM [K^+^]_o_ (de Paoli et al. 2007; Hansen et al. 2005; Pedersen et al. 2003). The protective effect on K^+^-depolarised muscle is due to partial inhibition of sarcolemmal ClC-1 channels since the effect is replicated with ClC-1 channel blockers, e.g. 9-anthracenecarboxylic acid (9-AC), and Cl^−^-free solutions (Fig. 10B; de Paoli et al. 2010; Pedersen et al. 2004, 2005). This protection does not involve [H^+^]_o_ since extracellular HCl did not restore force (Fig. 10A). These findings support the idea that H^+^/lactate^−^ protects against severe K^+^-induced force fatigue, given that K^+^-induced depolarisation can occur even during 30-s of muscular activity (Lindinger et al. 2024).

### Acidosis and inorganic phosphate

Interactions between P_i_ molecules and H^+^ on contraction are best studied using skinned muscle fibres where the internal milieu can be controlled. Nosek et al. (1987), obtained data on the H^+^–P_i_ interaction in skinned fast-twitch fibres (22 °C), and proposed [H_2_PO_4_^−^] to be a primary factor in force fatigue. Karatzaferi et al. (2008) later observed that reducing pH from 7.0 to 6.2 lowered maximum force to 97% initial, increasing [P_i_] from 5 to 30 mM (pH 7.0) reduced maximum force to 71% initial, and together at pH 6.2 + 30 mM P_i_ maximum force fell to 48% initial. This displays a synergistic effect of H^+^–P_i_ on peak force with these severe changes of pH and P_i_. Subsequent methodical work in the Fitts laboratory on skinned rat fibres (30 °C) showed a greater decline of maximum force at pH 6.2 + 30 mM P_i_ in fast-twitch type II fibres (with IIx > IIa) than in slow-twitch type I fibres (Fig. 9; Nelson and Fitts 2014; Nelson et al. 2014). These effects were synergistic, i.e., greater than simply being additive (Karatzaferi et al. 2008; Nelson and Fitts 2014; Nelson et al. 2014), and are consistent with the hypothesis that the reduced performance requires elevated [H_2_PO_4_^−^], rather than just raised [H^+^]_i_. However, the H^+^–P_i_ interaction on maximum force in skinned rabbit muscle fibres at lowered temperatures of 10–15 °C is shown to be additive rather than synergistic (Chase and Kushmerick 1988; Karatzaferi et al. 2003; Potma et al. 1995). Furthermore, at pH 6.2 the V_max_ fell to 72% initial, then with increasing [P_i_] to 30 mM the V_max_ recovered to 82% initial (Karatzaferi et al. 2008), which indicates that P_i_ protects against the acidosis effect to slow velocity. More recent work on skinned fibre segments from human vastus lateralis muscle (30 °C), found that at pH 6.2 + 30 mM P_i_ there was a 21% decline of maximum force, 11% decline of V_max_, and ~45% decline of peak power in type I fibres, of both young and older men (Sundberg et al. 2018). These depressive effects also manifested with smaller and graded H^+^–P_i_ perturbations, i.e., pH 6.8 + 12 mM P_i_ through to pH 6.2 + 30 mM P_i_, in skinned human muscle fibres although at 15 °C (Sundberg et al. 2024). Thus, human H^+^–P_i_ data replicates findings on force/power from animals. Combined data show that pH 6.2 + 30 mM P_i_ reduces peak power by 51% (range 42–67%).

### Acidosis and other fatigue factors

There are likely more H^+^ interactions to be discovered and studied. Interactions between K^+^ with lowered Na^+^-gradients (Cairns et al. 2022; Overgaard et al. 1999), lowered glycogen (Cairns and Renaud 2023), or altered [Ca^2+^]_o_ (Cairns et al. 2015) are all likely to modulate K^+^-H^+^ effects. Moreover, adrenaline and ß-agonists restore K^+^-depressed force (Bandschapp et al. 2012; Hansen et al. 2005; Pedersen et al. 2003) and when combined with acidosis exerts even greater protective effects (de Paoli et al. 2007; Pedersen et al. 2003; Hansen et al. 2005). When phosphorylation of myosin light chains in fast-twitch fibres is included then V_max_ is further depressed with acidosis (Cooke 2007; Karatzaferi et al. 2008), and this likely modifies the H^+^–P_i_ interaction. Similarly, lowered [PCr] increases maximum force (Fryer et al. 1995; Godt and Nosek 1989) and may resist H^+^–P_i_ effects on force. Also, elevated ROS impairs maximum myofilament function (Cooke 2007; Dutka et al. 2012), exacerbates trans-sarcolemmal ionic shifts with exercise (McKenna et al. 2006) and impairs T-system excitability (Watanabe and Wada 2020). Indeed, ROS interacts with H^+^ to depress submaximal contractions in rat diaphragm (Lawler et al. 1997).

### Physiological role of multiple interactions with acidosis during fatigue

There is no strong reason to discard any of the previously described interactions with H^+^ during fatigue. Now an important question is which interactive process dominates to influence symptoms during fatigue? We surmise that a detrimental H^+^–P_i_ effect would manifest early during fatiguing exercise when [P_i_] had increased (Fig. 3B, Dahlstedt et al. 2000). This interaction is likely to occur in temporal alignment with the early effects of induced acidosis on the fatigue profile (Feng and Jun 2016; Fig. 8B, C). The H^+^–K^+^ interaction may coexist with the H^+^–P_i_ interaction during fatigue, so that when K^+^ disturbances become larger an acidosis may combat excessive force loss to some extent. We propose that a H^+^–P_i_ effect on the myofilaments dictates and contributes to force depression, especially with the large acidosis in fast-twitch fibres. This H^+^–P_i_ effect via the myofilaments occurs later in the chain of events leading to contraction and hence would likely over-ride any improvement of SR Ca^2+^ release via the H^+^–K^+^ interaction which occurs earlier in the sequence. However, there is no direct evidence for effects of acidosis on cross-bridges dominating over excitability since experiments combining effects of H^+^, K^+^ and P_i_ on muscle have not been done.

## Peripheral mechanisms for effects of intracellular acidosis and lactate^−^

**Fig. 11:**
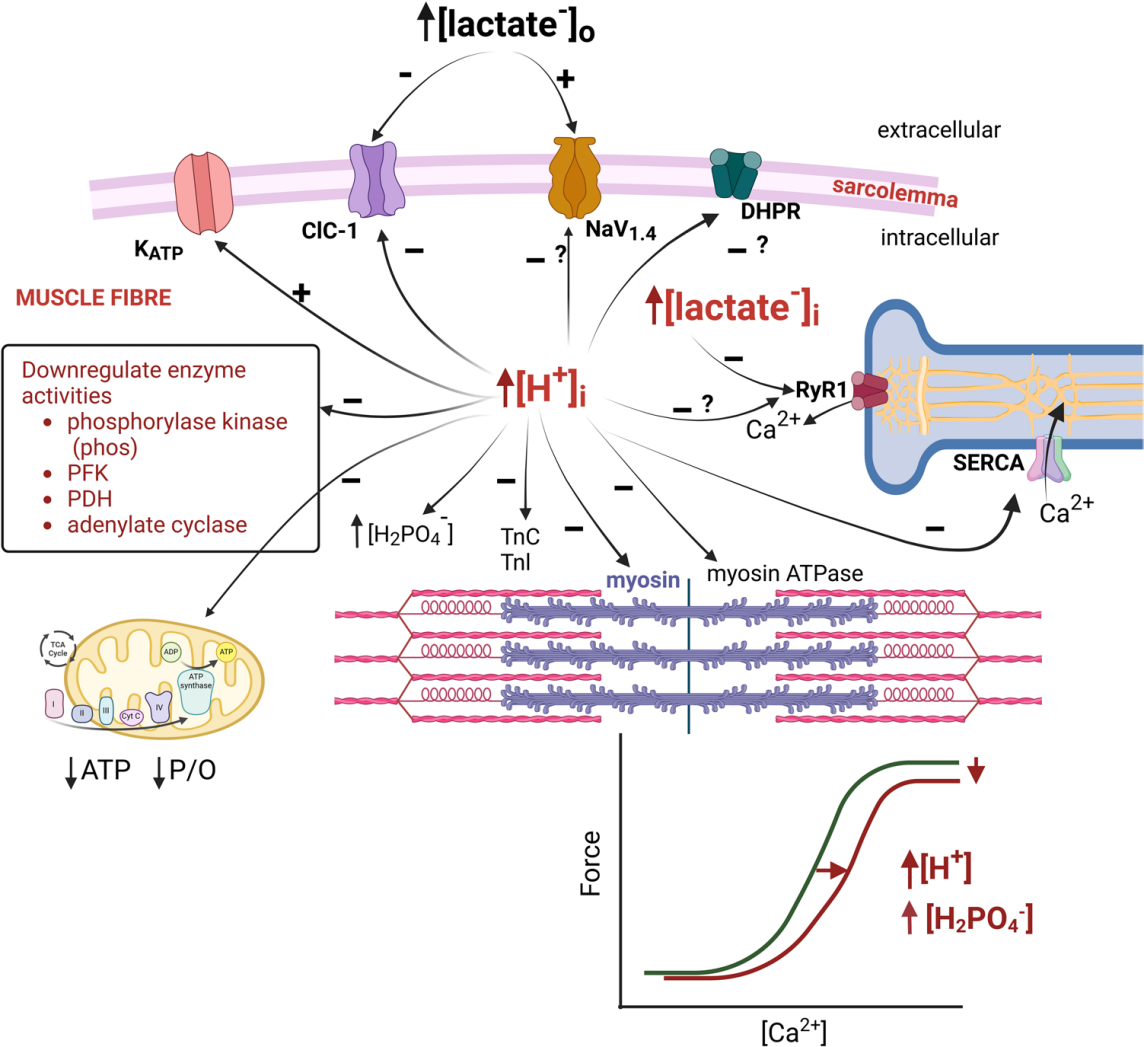
Schematic presentation of a muscle fibre with possible peripheral sites of modulation with raised [H^+^]_i_ or raised [lactate^−^]. ClC-1, sarcolemmal chloride channel; DHPR, voltage-sensor of excitation–contraction coupling; H_2_PO_4_^−^, diprotonated phosphate; K_ATP_, ATP-sensitive potassium channel; PDH, pyruvate dehydrogenase; PFK, phosphofructokinase; P/O, ratio of ADP phosphorylated to oxygen atom consumed; NaV1.4, voltage-activated sodium channel; RyR1, calcium release channel of sarcoplasmic reticulum (ryanodine receptor); SERCA, calcium pump of sarcoplasmic reticulum; TnC, troponin C; TnI, troponin I. Created using Biorender

Recent research has evolved the mechanistic details for effects of H^+^/lactate^−^ on cell processes, especially with studies utilising single muscle fibres from humans, and isolated myofilament proteins (for review see Debold and Westerblad 2024). We now appraise these mechanisms (Fig. 11, Table 3) and indicate what we consider to be the most physiologically relevant for human muscle fatigue.

**Table 3:** Postulated mechanisms for impairment of muscle contraction with H^+^ or lactate^−^

**Myofilament function**
Actomyosin cross-bridge activity
– ↓ maximum Ca ^2+^-activated force (H^+^, H_2_PO4^−^, lactate^−^, ↓ force)
– ↓ rate of cross-bridge detachment (H^+^, ↓ relaxation rate)
– ↓ myosin ATPase activity (H^+^, ↓ shortening velocity)
Regulatory proteins (troponin-C, troponin-I)
– ↓ Ca^2+^ sensitivity (H^+^, H_2_PO4^−^, ↓ submaximal force)
**T-system and sarcoplasmic reticulum**
– ↓ asymmetric charge movement (H^+^, no change)
– ↓ rate of SR Ca^2+^ release via ryanodine receptor channel (CICR) (H^+^, lactate^−^, ↓ force)
– ↓ rate of SR Ca^2+^ uptake via SERCA (i.e., ↓ Ca^2+^-ATPase activity) (H^+^, ↓ relaxation rate)
**Sarcolemma and action potentials**
– ↑ action potential amplitude and excitability (H^+^, lactate^−^, force restoration)
– ↓ action potential conduction velocity (H^+^)
– ↑ Sodium (NaV1.4) channel conductance (H^+^, lactate^−^, force restoration)
– ↓ ClC-1 channel conductance (H^+^, lactate^−^, force restoration)
– ↑ K_ATP_ channel conductance (H^+^, force restoration)
– ↑ Na^+^-K^+^- ATPase activity (H^+^ via ↑ [Na^+^]_i_, force restoration)
**Metabolism**
– ↓ Rate of ATP supply to ↓ [ATP]_i_ (H^+^, HATP^3−^, ↓ power)
– ↓ PFK, ↓ Phos, ↓ PDH activities
– ↓ adenylate cyclase activity (↓ cAMP)
– ↓ PCr
– ↓ Mitochondrial oxidative capacity and/or ↓ mitochondrial efficiency
– ↓ ROS or ↑ ROS – H^+^ interaction

*cAMP* cyclic adenosine monophosphate, *CICR* calcium induced calcium release, *K*_*ATP*_ ATP dependent potassium channel, *NaV1.4* voltage activated sodium channel, *PDH* pyduvate dehydrogenase, *PFK* phosphofructokinase, *Phos* glycogen phosphorylase, *ROS* reactive oxygen species, *SERCA* Ca^2+^-ATPase activity of sarcoplasmic reticulum

### Myofilament function

The maximum Ca^2+^-activated force falls by up to 15% initial when pH_i_ is lowered to 6.7–6.2 at physiological temperatures in both intact (Westerblad et al. 1997; Westerblad and Allen 1993) and skinned mammalian fibres (Karatzaferi et al. 2008; Knuth et al. 2006; Lamb and Stephenson 1994; Nelson and Fitts 2014; Pate et al. 1995). This manifests as a downwards shift of the sigmoidal force-[Ca^2+^]_i_ relationship (Fig. 11). In contrast to animal studies no significant decline of maximum force was detected in human fibres (Lynch et al. 1994; Olsson et al. 2020). Similarly, raised [lactate^−^] exerted only minor effects on maximum force in skinned mammalian fibres (Andrews et al. 1996; Dutka and Lamb 2000; Posterino and Fryer 2000; Posterino et al. 2001). The severe but physiologically important combination of pH 6.2 + 30 mM P_i_ (30 °C) lowered maximum force by 41% (range 20–52%) in skinned mammalian and human fibres (Karatzaferi et al. 2008; Nelson and Fitts 2014; Nelson et al. 2014; Sundberg et al. 2018). Smaller decrements have recently been shown for lesser physiological H^+^–P_i_ perturbations in human muscle fibres (15 °C) (Sundberg et al. 2024). Sophisticated experiments on isolated myosin molecules, using a mini-ensemble laser trap assay, demonstrated that the force generating capacity falls by ~20% at pH 6.5 (Woodward and Debold 2018). They found that acidosis reduced the force per cross-bridge via a slowed rate of myosin attachment to actin, and some non-productive interactions that generate negative forces. Raised [P_i_] also lowers maximum force (Debold et al. 2006; Fryer et al. 1995; Karatzaferi et al. 2003) although by a mechanism which is distinct to that of acidosis: this P_i_ effect involves accelerating myosin detachment from actin (Debold et al. 2013; Woodward and Debold 2018). Hence, raised [H^+^] and [P_i_] act by separate molecular process but whether they are additive or synergistic requires further research.

A marked induced acidosis to pH 6.5–6.0 reduced muscle shortening velocity by 15–30% (Karatzaferi et al. 2008; Knuth et al. 2006; Nelson and Fitts 2014), which is likely consequent to a reduced myosin ATPase activity (Blanchard et al. 1984; Parkhouse 1992; Woodward and Debold 2018). Raised [lactate^−^] is without effect on myosin ATPase activity (Parkhouse 1992). When utilising the in vitro actin motility assay an imposed acidosis to pH 6.8–6.2 reduced the myosin driven unloaded actin filament velocity via a slowed myosin detachment from actin (Debold et al. 2008, 2012; Greenberg et al. 2010; Jarvis et al. 2018; Longyear et al. 2014; Woodward and Debold 2018). This mechanism explains how raised [H^+^]_i_ slows unloaded shortening velocity. In contrast, 30 mM [P_i_] prompted an increased actin sliding velocity and more rapid myosin detachment under acidosis conditions (Debold et al. 2011, 2013; Longyear et al. 2014). Elevated [P_i_] at low pH (and hence raised [H_2_PO_4_^−^]) also increased myosin ATPase activity (Greenberg et al. 2010; Jarvis et al. 2018; Woodward and Debold 2018). These findings explain why raised [P_i_] per se does not reduce muscle shortening velocity (Fig. 9). Interestingly, myosin phosphorylation caused a slowing of actin sliding (Cooke 2007; Greenberg et al. 2010; Longyear et al. 2014) to worsen the acidosis-induced reduction of shortening velocity in fast-twitch fibres (Karatzaferi et al. 2008). This process may be an important physiological interaction in fast-twitch muscle fibres during exercise.

A dominant effect of acidosis entails a reduced myofilament Ca^2+^-sensitivity, seen as a rightwards shift of the force-[Ca^2+^]_i_ relationship in rodent (Fig. 11; Nelson et al. 2014; Parsons et al. 1997; Pate et al. 1995; Westerblad et al. 1997; Westerblad and Allen 1993) and human fibres (Lynch et al. 1994; Olsson et al. 2020). This shift is greater in fast-twitch than slow-twitch fibres (Lynch et al. 1994; Nelson and Fitts 2014) and can explain the large suppression of submaximal force at lowered [Ca^2+^]_i_ (Adams et al. 1991; Harkema and Meyer 1997; Harkema et al. 1997; Meyer et al. 1991). Raised [lactate^−^] has little or no effect on Ca^2+^-sensitivity (Andrews et al. 1996; Dutka and Lamb 2000; Posterino et al. 2001). Reducing pH to 6.5 competitively inhibits Ca^2+^ binding to troponin-C (TnC) mainly due to direct actions on troponin-C (El-Saleh and Solaro 1988; Parsons et al. 1997; Unger and Debold 2019) but also via lowered affinity of troponin-I (TnI) for troponin-C (El-Saleh and Solaro 1988; Robertson et al. 2012). Raised [P_i_] also reduces Ca^2+^-sensitivity in skinned fibres (Debold et al. 2006; Fryer et al. 1995) to exacerbate this effect at lowered pH (Nelson and Fitts 2014). This impaired Ca^2+^-sensitivity markedly reduces force and power at sub-saturating [Ca^2+^]_i_ (Nelson and Fitts 2014), a condition which occurs with impaired Ca^2+^ release during fatigue (Allen et al. 2008; Dahlstedt et al. 2000).

### Ca^2+^ handling by sarcoplasmic reticulum

Acidosis may, in principle, impair excitation–contraction coupling resulting in less Ca^2+^ release from the SR (Fig. 11, Table 3). However, a CO_2_-induced acidosis (pH_i_ 6.7) elevated rather than a depressed tetanic [Ca^2+^]_i_ in non-fatigued rodent (Westerblad and Allen 1993) and human fibres (Olsson et al. 2020). This negates the hypothesis that acidosis impairs SR Ca^2+^ release in intact fibres. Moreover, T-tubular membrane charge movement, signifying dihydropyridine receptor (DHPR) activity, i.e. function of the voltage sensor of excitation–contraction coupling (Fig. 11), was unaffected at pH_i_ 6.2 in amphibian fibres (Balog and Fitts 2001). When acidification (pH 6.6–6.5) was tested more specifically on Ca^2+^ release channels (i.e., RyR1) in isolated SR vesicles or lipid bilayers then Ca^2+^ release was impaired (Favero et al. 1995; Laver et al. 2000; Rousseau and Pinkos 1990). In these experiments SR Ca^2+^ release was activated by Ca^2+^ or ATP rather than normal physiological voltage activation. When action potentials were employed, Ca^2+^ release was unhindered at pH_i_ 6.2 in skinned rat EDL fibres (Lamb and Stephenson 1994). Similarly, 25–30 mM [lactate^−^] inhibited Ca^2+^-induced Ca^2+^ release (CICR) (Dutka and Lamb 2000; Favero et al. 1995; Spangenburg et al. 1998) but with minimal effect on SR Ca^2+^ release (~5% decline) when triggered with action potentials (Dutka and Lamb 2000). Therefore, H^+^/lactate^−^ does not impair SR Ca^2+^ release when normal physiological processes are involved. Lastly, a CO_2_-induced acidosis slowed both the decline of [Ca^2+^]_i_ and mechanical relaxation after a brief tetanus in mouse and human fibres (Olsson et al. 2020; Westerblad and Allen 1993). This aligns with a slowing of Ca^2+^ uptake by the SR Ca^2+^-pump (SERCA) since acidosis inhibits its activity at both maximal and submaximal [Ca^2+^] (MacLennan 1970; Wolosker et al. 1997). Hence, H^+^-inhibition of SERCA likely contributes to slower relaxation.

### Action potentials

Neither lactate^−^ or acidosis influence the resting membrane potential (Erdoğan et al. 2002; Hansen et al. 2005; Juel 1988b; Pedersen et al. 2005). However, intracellular acidosis evokes a small increase of action potential peak and maximal rate of rise of the action potential, a lowered rheobase (Lehmann-Horn et al. 1987; Pedersen et al. 2005), and slowed action potential conduction velocity (Brody et al. 1991; Juel 1988b). None of these effects reduce force to cause fatigue. Also in K^+^-depressed rodent fibres, an induced acidosis prompts recovery of M-wave amplitude (de Paoli et al. 2007; Hansen et al. 2005; Nielsen et al. 2001; Pedersen et al. 2003, 2005) an increased intracellular action potential amplitude, and greater number of excitable fibres (Pedersen et al. 2005). These effects can all be explained by a lowered ClC-1 conductance (Pedersen et al. 2005). Na-lactate exposure has been shown to increase Na^+^-K^+^-ATPase activity in resting fibres, presumably due to raised [Na^+^]_i_ (Kristensen et al. 2005), but this is not always seen (de Paoli et al. 2007). Myoplasmic acidosis inhibits isolated ClC-1 channel activity (Bennetts et al. 2007), increases K_ATP_ channel activity (Renaud et al. 2023; Xu et al. 2001) and enhances currents through voltage-activated Na^+^-channels (NaV1.4) in human muscle (Lehmann-Horn et al. 1987). Furthermore, 5–20 mM [lactate^−^]_o_ per se restored the M-wave in K^+^-depressed fibres by reducing ClC-1 channel conductance (de Paoli et al. 2010), and 10 mM [lactate^−^] directly increased maximal Na^+^-currents which may be beneficial in K^+^-depressed conditions (Rannou et al. 2012). Hence, acidosis/lactate^−^ preserves excitability and action potential amplitude under depolarised conditions in resting muscle (Pedersen et al. 2005) to maintain Ca^2+^ release (Wang et al. 2022). More work is needed to understand the K^+^-H^+^ effects in relation to fatigue.

### Metabolism

Intracellular acidosis may influence muscle contractile function indirectly via effects on enzymes or mitochondria to reduce ATP supply (Fig. 11). Acidosis severely inhibits phosphofructokinase (PFK) and glycogen phosphorylase (Phos) activities in vitro (Dobson et al. 1986; Kasvinsky and Meyer 1977; Trivedi and Danforth 1966). The protonated version of ATP, i.e. HATP^3−^, is a potent inhibitor of PFK (Dobson et al. 1986; Sahlin 1983). However, with in situ conditions the various enzyme activators better maintain PFK activity (Dobson et al 1986; Sutton et al. 1981). Phos activity is inhibited with exercise-induced acidosis (hence lesser conversion of Phos b to the active Phos a isoform) (Chasiotis et al. 1982; Hollidge-Horvat et al. 1999; Howlett et al. 1998; Parolin et al. 1999). Acidosis also reduces adenylate cyclase activity in vivo to lower cyclic adenosine monophosphate (cAMP) levels, i.e., an important activator of Phos b (Chasiotis et al. 1982). Reduced cAMP levels may also impact force via excitation–contraction coupling (Cairns and Borrani 2015). Despite the exercise-induced downregulation of these enzymes at pH_i_ 6.8–6.6 the glycogenolytic/glycolytic rates still exceed 60% of initial in contracting quadriceps muscle (Bangsbo et al. 1996; Chasiotis et al. 1987; Kemp et al. 2001; Sahlin et al. 1975; Spriet et al. 1987a, 1989). Moreover, a NH_4_Cl-induced acidosis during exercise lowers activation of pyruvate dehydrogenase (PDH) (Hollidge-Horvat et al. 1999). Therefore, acidosis lowers lactate^−^ production, glycogen depletion and pyruvate oxidation (Fig. 5; Hollidge-Horvat et al. 1999; Parolin et al. 1999; Spriet et al. 1987a, 1989; Sutton et al. 1981). Acidosis also slows the creatine kinase reaction (Conley et al. 2001; Sahlin et al. 1975) to lower [PCr]_i_ at rest (Sahlin et al. 1983) or during exercise (Churchward-Venne et al. 2010; Sahlin et al. 1975).

An intracellular acidosis inhibits oxidative phosphorylation during human exercise (Conley et al. 2001; Robergs et al. 2004) due to a lowered mitochondrial oxidative capacity (Bartlett et al. 2020, 2021; Jubrias et al. 2003; Layec et al. 2013; Walter et al. 1997) and/or reduced mitochondrial efficiency, i.e. decreased P/O ratio (Broxterman et al. 2017a, b). In line with this, an induced acidosis reduces oxidative capacity in cat soleus in situ (Harkema and Meyer 1997) and in skinned rat fibres (when P_i_ is also increased) (Walsh et al. 2002) and reduces efficiency in isolated mitochondria from mice (with increased temperature) (Flensted-Jensen et al. 2024). Moreover, acidosis reduces some mitochondrial respiratory complex activities along with attenuating ROS production (Hedges et al. 2019). Different effects of acidosis on mitochondrial function between studies may arise through different muscle types studied, types of preparation, and degree of acidity (Layec et al. 2013). Such effects require further research.

The combined effects of acidosis on metabolism lowers the rate of ATP supply, (Bartlett et al. 2020, 2021; Robergs et al. 2004) with bulk [ATP]_i_ falling but by less than 20% initial (Bartlett et al. 2020; Black et al. 2017; Chasiotis et al. 1987; Newham and Cady 1990; Parolin et al. 1999; Spriet et al. 1989; Vigh-Larsen et al. 2022). Hence [ATP]_i_ is largely maintained in exercising muscle when a reduced ATP supply is matched by reduced ATP demand by physiological processes that lower force/power (Broxterman et al. 2017a, b).

#### Summary

Acidosis to pH_i_ 6.5–6.2 impairs myofilament function via a reduced maximal force, myosin ATPase activity and Ca^2+^-sensitivity. These effects together lower force, shortening velocity and peak power. Acidosis also slows myofilament sliding and reduces SERCA activity to prolong relaxation. Raised H^+^/lactate^−^ ameliorates harmful effects of K^+^ on action potentials, via ClC-1 and NaV1.4 channels to counter extreme force fatigue. Acidosis inhibits glycogenolytic/ glycolytic enzyme activity and mitochondrial function to reduce ATP generation.

## Role of extracellular acidosis/lactate^−^ in central fatigue and exercise performance

**Fig. 12:**
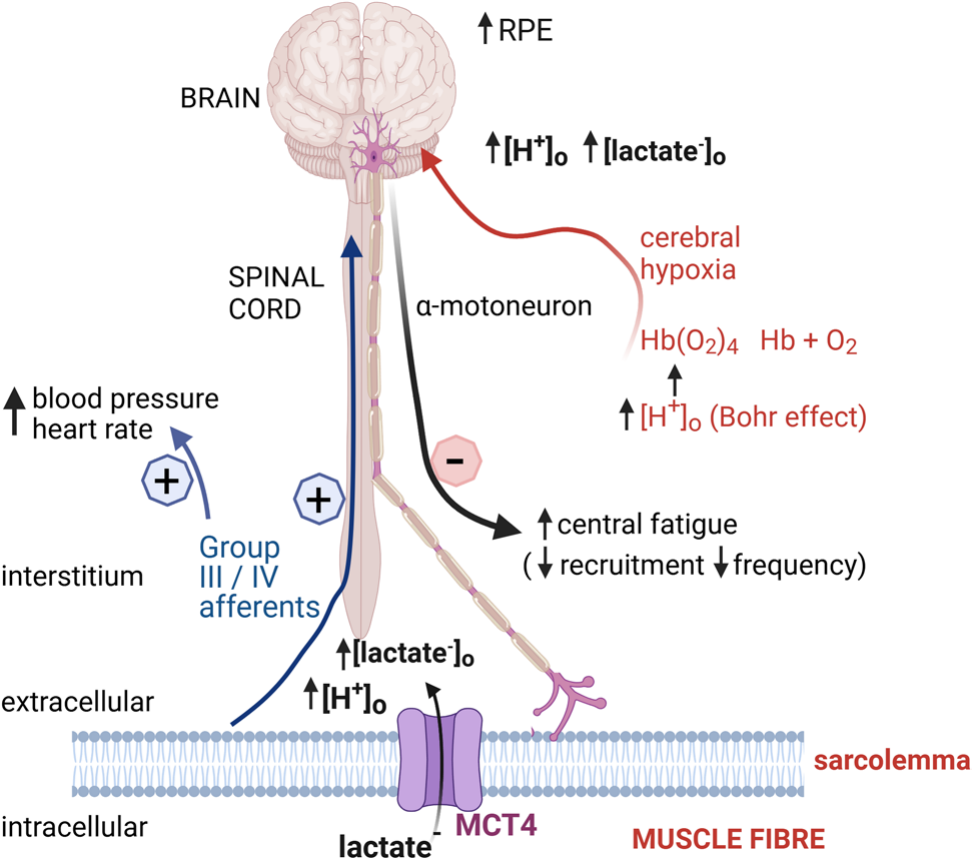
Schematic presentation of the central nervous system (brain, spinal cord) with a muscle fibre, and possible central sites of modulation with raised [H^+^]_o_ or [lactate^−^]_o_. This may provoke central fatigue (diminished recruitment, lowered motoneuron firing frequency) or heighten ratings of perceived exertion (RPE). Possible mechanisms include: increased firing of group III/IV muscle afferents; severe Bohr effect leading to cerebral hypoxia; direct cerebral effects of circulating H^+^/lactate^−^. Created using Biorender

It has been hypothesized that elevated H^+^/lactate^−^ may impair exercise performance via the central nervous system (CNS) with reduced voluntary activation and/or heightened fatigue sensations (Fig. 12; Cairns 2006; Hureau et al. 2022; Siegler and Marshall 2015). Indeed, Kent-Braun (1999) found that with fatigue during a prolonged MVIC of ankle dorsi-flexor muscles, the voluntary activation ratio fell from 0.94 to 0.78, i.e., central fatigue occurred, and there was an association between pH_i_ and integrated electromyogram. Hureau et al. (2022) also found a fall in voluntary activation ratio from 0.88 to 0.73 during repeated MVIC of knee extensors that was linearly correlated with an increase of [H^+^]_i_. Both studies report an associative correlation between pH_i_ and central fatigue, yet this is not evidence for cause and effect. In fact, a protuberant feature of the latter study was that at 5 min post-exercise the pH_i_ remained low at 6.7, yet the voluntary activation ratio had returned to normal. This observation indubitably challenges their correlative prediction and testifies that an intracellular acidosis of this magnitude does not cause central fatigue. It should be emphasized that a detrimental effect of acidosis via the CNS requires an extracellular rather than intracellular acidosis, with H^+^-induced feedback via sensory afferents or increased plasma or cerebrospinal fluid [H^+^] acting directly or indirectly on the brain (Fig. 12).

With intense exercise pH_o_ falls markedly in venous plasma draining contracting muscle (Table 1) but is not correlated with loss of peak power during cycling (Mildenhall et al. 2023). The pH_o_ in muscle interstitium also falls from 7.4 to 7.0 or less (Steinhagen et al. 1976; Street et al. 2005) and this location is where group III/IV afferents are found (Molliver et al. 2005; Pollak et al. 2014). Infusion of lactate^−^, with lowered pH, and ATP into the interstitium of human adductor pollicus muscle evoked fatigue sensations with each molecule individually being without effect (Pollak et al. 2014). Maximum fatigue sensations occurred at pH_o_ 7.2, 10 mM [lactate^−^]_o_, and 500 nM ATP, with higher levels of these metabolites causing pain. Furthermore, greater acidification with ingested NH_4_Cl during a 4 km cycle time-trial elevated RPE (Correia-Oliveira et al. 2017) and fatigue sensations during high-intensity submaximal exercise (Kostka and Cafarelli 1982). Conversely, alkalosis with ingested NaHCO_3_ attenuated the rise of RPE during intense incremental exercise (Krustrup et al. 2015; Swank and Robertson 1989, 1986. These findings confirm that extracellular acidosis contributes to fatigue sensations and elevated RPE, but whether extracellular acidosis evokes central fatigue is not established. Alkalosis with NaHCO_3_ does not recover voluntary activation of leg muscles during fatigue (Siegler et al. 2016; Siegler and Marshall 2015). The sole piece of direct evidence that extracellular acidosis evokes central fatigue is when voluntary activation was 19% higher with NaHCO_3_ than control during a post-fatigue ischaemic period (Siegler and Marshall 2015).

Three potential mechanisms exist by which elevated [H^+^]_o_/[lactate^−^]_o_ may contribute to central fatigue (Fig. 12). These include sensory feedback via muscle group III/IV afferents to the CNS, O_2_ desaturation of haemoglobin leading to severe cerebral hypoxia, and direct effects of circulating H^+^/lactate^−^ on the brain.

### Muscle group III/IV afferent feedback

Amann and colleagues have tested the role of muscle sensory feedback during intense exercise, using lumbar intrathecal fentanyl injection—this agent blocks group III/IV afferent firing. During repeated intermittent MVIC of quadriceps muscle, fentanyl attenuated the rise of RPE but not the impairment of peak force (Broxterman et al. 2017b, 2018). With intense cycling during a 5 km time-trial, or at constant workload, application of fentanyl exacerbated the decline of force and voluntary activation, and attenuated RPE early on but not at end-exercise (Amann et al. 2011; Blain et al. 2016). Hence, activation of group III/IV afferents elevates RPE for single muscle group contractions and early into whole-body exercise. Since activation of these afferents did not impair exercise performance it is reasoned that effects of H^+^/lactate^−^ on these afferents would not promote central fatigue. Furthermore, studies using anaethetised animals report increased firing of group III/IV afferents with exogenous application of lactic acid with maximal effects occurring at 1 mM (Caron et al. 2015; Darques et al. 1998; Decherchi et al. 1998). Afferent firing was attenuated when H^+^/lactate^−^ production was pharmacologically suppressed during stimulation (Darques et al. 1998). Selective blockade of the acid-sensing ion channels found in sensory afferents of mice attenuated effects of lactic acid and ATP (Light et al. 2008). It is apparent that low concentrations of lactate^−^/H^+^ are sufficient to trigger group III/IV afferents which presumably brings the exercise pressor reflex into play (Boushel et al. 1998; MacLean et al. 2000).

### Plasma acidosis

A plasma acidosis can lower the affinity of haemoglobin for O_2_ resulting in arterial O_2_ desaturation (i.e. fall of SaO_2_), via the Bohr effect (Nielsen 2003; Nielsen et al. 2002a). In consequence, lower cerebral O_2_ delivery may be severe enough to cause central fatigue (Nielsen et al. 1999; Nybo and Rasmussen 2007). Thorough research by Nielsen et al. (2002a) showed that with simulated rowing, a fall of pH_o_ (7.42–7.07) occurred alongside a decreased S_a_O_2_ (from 97.5 to 89.0%). When the same rowers had NaHCO_3_ infused there was a lesser fall of pH_o_, (to 7.35), a higher S_a_O_2_ (94%), and improved performance time. These findings align with plasma H^+^ causing the unloading of O_2_ from haemoglobin to severely lower cerebral O_2_ levels (Nielsen et al. 1999) which may cause central fatigue. Indeed, an experimentally lowered S_a_O_2_ per se has been shown to impair intense exercise performance (Nielsen et al. 1999; Nybo and Rasmussen 2007). With world-class cyclists, ingestion of NaHCO_3_ was without effect on S_a_O_2_ and peak power during a maximal test (Mildenhall et al. 2023). However, arterial desaturation may feature more in rowing where plasma acidosis is often extreme (Nielsen 1999; Nielsen et al. 1999, 2022a; b; Table 2). The third and simple notion of direct harmful effects of plasma H^+^/lactate^−^ acting on the brain appears to be unsupported. An opposing argument to this notion, is that lactate^−^ is an established fuel for many cell types in the brain which is beneficial (Brooks 2018; Ferguson et al. 2018; Quistorff et al. 2008).

#### Summary

There is little direct support for the hypothesis that extracellular acidosis causes central fatigue during exercise. The possibility remains that it contributes when there is an extreme plasma acidosis and arterial desaturation of haemoglobin.

## Manipulation of H^+^/lactate^−^ regulation and muscle/exercise performance

Experimental exploitation of H^+^/lactate^−^ regulatory processes in muscle or plasma has the potential to shed considerable light on the role of both ions in fatigue (Fig. 6).

### Sodium bicarbonate and sodium citrate as extracellular H^+^ buffers

Some of the most compelling evidence to support the role of acidosis in fatiguing human exercise comes from studies using NaHCO_3_ (typically ~0.3 g/kg body mass) or Na-citrate (typically ~0.5 g/kg body mass). These extracellular H^+^-buffers often enhance performance during high-intensity exercise of 1–10 min duration. There are well documented performance improvements for running (Bird et al. 1995; Krustrup et al. 2015; van Montfoort et al. 2004), rowing (Boegman et al. 2020; Nielsen et al. 2002a) and cycling (Costill et al. 1984; Gough et al. 2018; Messonnier et al. 2007). Time-trial performance during sport racing events of 4–7 min is improved slightly but importantly by 2–9 s (Bird et al. 1995; Gough et al. 2018; Nielsen et al. 2002a). Despite this, ergogenic effects are not always seen given that the effect depends on the extent of plasma HCO_3_^−^ loading, exercise regime, abundance of H^+^/lactate^−^ regulatory proteins, and training status (de Oliveira et al. 2022; Messonnier et al. 2007). NaHCO_3_ or Na-citrate intake normally raises pre-exercise plasma [HCO_3_^−^] from ~25 to 30–35 mM which attenuates the fall of plasma and interstitial pH with intense exercise (Correia-Oliveira et al. 2017; Gough et al. 2018; Mildenhall et al. 2023; Nielsen et al. 2002a; Street et al. 2005). Although HCO_3_^−^ is thought not to enter the myoplasm, the elevated [Na^+^]_o_ and [HCO_3_^−^]_o_ better maintains the trans-sarcolemmal [H^+^]-gradient to facilitate lactate^−^ extrusion. This in turn can reduce the exercise-induced intracellular acidosis, with the HCO_3_^−^-effect being up to 0.2 pH units, e.g., from pH_i_ 6.4 to 6.6 (Costill et al. 1984; Nielsen et al. 2002b; Raymer et al. 2004; Stephens et al. 2002). This pH_i_ recovery is of sufficient magnitude to enhance force/power (Fig. 3). Raised [NaHCO_3_]_o_ does not alter pH_i_ during submaximal or brief maximal exercise (Nielsen et al. 2002b), with stimulation regimes evoking moderate acidosis (e.g., pH_i_ 6.8) (Broch-Lips et al. 2007), or at termination of more prolonged intense exercise (Costill et al. 1984; Raymer et al. 2004). Exactly how NaHCO_3_ improves time-trial performance remains unclear since it sometimes but not always ameliorates loss of peak force/power (Grgic et al. 2020; Mildenhall et al. 2023; Siegler and Marshall 2015). The alkalinizing effects may also restore the rate of rise of force (Grgic et al. 2020) or improve cycling cadence (Mildenhall et al. 2023). Future work is needed to understand exactly how extracellular H^+^-buffers influence pH_i_ during exercise to better evaluate the role of H^+^ in performance.

### Monocarboxylate transporters (MCT)

The main regulators of lactate^−^ movement across the sarcolemma in mammalian skeletal muscle are MCT1 and MCT4 (Brooks 2018; Juel 1988a; Lindinger et al. 2013) (Figs. 1 and 6). The MCT4 isoform primarily extrudes lactate^−^ during exercise, especially for contracting fast-twitch fibres which generate large increases of [lactate^−^]_i._ The MCT1 isoform imports lactate^−^ most notably in quiescent slow-twitch fibres in non-contracting muscle (Kowalchuk et al. 1988b; Lindinger et al. 2013). These two MCT isoforms can therefore be used to explain the lactate shuttle theory (Brooks 2018). When fast-twitch FDB fibres were fatigued in vitro using low-intensity repeated tetani the pH_i_ was unchanged in control conditions but when lactate^−^ efflux via MCT was blocked with cinnamate (Fig. 6), the pH_i_ fell by 0.4 pH units and fatigue was more rapid (Westerblad and Allen 1992). This result is interpreted as inhibition of MCT4 leading to greater [lactate^−^]_i_ during stimulation, which then raises [H^+^]_i_ to exacerbate fatigue. Also, when mice undergo intense treadmill running during pharmacological blockade of MCT (Kitaoka et al. 2022) or global knockout of MCT4 (i.e. MCT4^−/−^) (Bisetto et al. 2019) there is a reduced time to exhaustion. When MCT activity was abolished, the working muscles had greater [lactate^−^]_i_ as predicted (Bisetto et al. 2019; Kitaoka et al. 2022). Unexpectedly, this effect was not seen with incremental running (Tamura et al 2024). Moreover, the fatigue profile of isolated fast-twitch muscles was unchanged with MCT4^−/−^ or partial MCT1, knockout (Bisetto et al. 2019; Chatel et al. 2017; Tamura et al. 2024). This implies that modulation of lactate^−^ transport at sites away from working muscle may impact exercise tolerance (Bisetto et al. 2019; Kitaoka et al. 2022). More research is needed to clarify these differences with genetic modification of the MCT isoforms.

### Carnosine and carbonic anhydrase

Greater abundance of muscle H^+^-regulatory proteins is correlated with a lesser myoplasmic acidosis and better performance during intense exercise (Messonnier et al. 2007). These regulatory processes which include intracellular H^+^-buffers (carnosine^−^, PCr, P_i_, HCO_3_^−^, histidine residues on proteins) (Figs. 1 and 6), carbonic anhydrase and NHE, have all been studied in relation to training and fatigue (Gunnarsson et al. 2013; Hostrup et al. 2021; Juel et al. 2004). NHE is required for pH_i_ recovery after exercise rather than during repeated contractions (Juel 1988a). We now focus specifically on the roles of carnosine and carbonic anhydrase during exercise.

Muscle carnosine is a dipeptide which acts as a myoplasmic H^+^-buffer, albeit with effects on Ca^2+^ handling, myofilament Ca^2+^-sensitivity, and ROS (Allen et al. 2008; Matthews et al. 2019). Several reviews report that chronic β-alanine supplementation augments muscle carnosine levels and buffer capacity (Fig. 6) in a manner thought to provide resistance to acidosis and fatigue (Matthews et al. 2019; Saunders et al. 2017). Such supplementation exerts small ergogenic effects (Baguet et al. 2010; Derave et al. 2007; Hill et al. 2007; Matthews et al. 2019) although they are not consistently observed (Black et al. 2018; Derave et al. 2007).

Carbonic anhydrase (CA) catalyses’ the reversible reaction: H^+^  + HCO_3_^−^ ↔ H_2_CO_3_ ↔ CO_2_ + H_2_O. The abundance of CA isoforms correlates with the fall of pH_i_ during supramaximal cycling implicating that it protects against acidosis (Messonnier et al. 2007). Moreover, acute or chronic use of acetazolamide, an inhibitor of CAI, evokes an extracellular metabolic acidosis and markedly reduces exercise time to exhaustion in humans (Doherty et al. 2023; Gonzales and Scheuermann 2013; Kowalchuk et al. 2000). Knock-out or overexpression of CAIII modifies the fatigue profile during repeated tetani in rodent muscle (Liu et al. 2007). Gastrocnemius muscle of CAIII knock-out mice display a slightly greater acidosis, i.e., pH_i_ 6.55 versus 6.65, over a 2-min stimulation period. Tibialis anterior muscle of CAIII knock-out mice had an initial more rapid fatigue profile (Feng and Jin 2016). In contrast, with soleus muscles of knock-out mice the fatigue profile was unchanged despite a higher [lactate^−^]_i_ (Feng and Jin 2016). Hence CAIII activity is protective for the initial decline of force during fatiguing stimulation but only in fast-twitch muscle.

#### Summary

Supplementation with extracellular H^+^-buffers permits small improvements in performance times together with a slightly lesser intracellular acidosis that seems to cause these effects. Reducing sarcolemmal MCT1 and MCT4 activity impairs exercise tolerance, causing a higher [lactate^−^]_i_ (and presumably lower pH_i_) yet may not directly impair muscle performance. The role of β-alanine, carnosine and CA needs more research along with measurement of pH_i_.

## Lactic acidosis and fatigue: Current state of understanding

The question posed of whether lactic acid or acidosis is the “major factor in fatigue” needs to be redefined with two questions being addressed. First, “Does raised lactate^−^/acidosis have a large detrimental effect on muscle performance during human exercise?” This will answer whether lactate^−^/acidosis is a direct cause or indirect contributor to considerable muscle fatigue. Second, “Does lactate^−^/acidosis have a functionally important effect on exercise performance during human exercise?” This would decipher whether lactate^−^/acidosis causes impairment or protection of exercise performance regardless of the magnitude of its effect.

We sum up the key points of the present review with our perspectives:

1. Accumulation of extracellular lactate^−^ (to 25 mM) or intracellular lactate^−^ (to 50 mM) (Table 1) has very little detrimental effect per se on muscle/exercise performance. However, lactate^−^ may contribute indirectly via metabolic acidosis.
2. Despite an intracellular acidosis occurring at the whole muscle level during intense exercise where pH_i_ falls from ~7.0 to 6.9–6.3 (Table 2, Figs. 2, 3 and 5), a large acidosis features only in fast-twitch fibres (to pH_i_ ~ 6.2) and not in slow-twitch fibres (to pH_i_ ~ 6.9). Hence, raised [H^+^]_i_ is a realistic putative fatigue factor only in fast-twitch fibres.
3. The peak power/force-pH_i_ (or [H^+^]_i_) relationship during fatiguing voluntary contractions in humans displays considerable variation with different exercise protocols (Fig. 3). This suggests that intracellular acidosis is not the sole cause of fatigue. Other putative fatigue factors (e.g., phosphate metabolites, fuel supply, trans-sarcolemmal ionic disturbances, ROS) also change during high-intensity exercise and may alter performance (Figs. 2 and 5).
4. Correlative associations between pH_i_ (or [H^+^]_i_) and the decline of force/power can lead to incorrect conclusions and do not prove cause and effect. Such studies should receive less emphasis with focus being put on experimental manipulation of pH/lactate^−^ regulatory processes.
5. Intracellular acidosis to pH_i_ ~ 6.7–6.6 (28–37 °C), has little depressing effect on maximum isometric force (<5% peak) when this acidosis occurs during fatiguing activity or recovery of human muscle, or when tested with an induced-acidosis in non-fatigued muscle. Hence, an intracellular acidosis of this magnitude per se would not cause much force fatigue.
6. A larger intracellular acidosis to pH_i_ 6.5–6.2 per se (30–37 °C) reduces maximum isometric force (~12% initial), shortening velocity (~5% initial), and muscle power (~22% initial) in non-fatigued muscle (Fig. 9). This acidosis also slows mechanical relaxation.
7. Ingestion of NH_4_Cl amplifies the exercise-induced intracellular acidosis (up to 0.2 pH units) which worsens exercise performance in humans during high-intensity cycling and rowing.
8. A pre-exercise induced acidosis (metabolic, respiratory) alters the fatigue profile during electrical muscle stimulation in humans and animals by accelerating the early loss of force/power (5–30% initial) (Fig. 8).
9. Increased [H^+^]_i_ during exercise either acts directly or contributes to fatigue through interactions with raised [P_i_] (and may require raised [H_2_PO_4_^−^]_i_) (Figs. 2, 4, 5 and 9).
10. Depressive effects of raised [H^+^]_i_ and [P_i_]_i_ (mainly raised [H_2_PO_4_^−^]_i_) on force occur primarily via myofilament proteins to reduce maximum cross-bridge function and Ca^2+^-sensitivity. In addition, raised [H^+^]_i_ reduces shortening velocity via lowered myosin ATPase activity.
11. Intracellular acidosis (and increased [lactate^−^]_o_) protects against depressive effects of raised [K^+^]_o_ on action potentials and force in resting muscle (Fig. 10). This involves a reduced ClC-1 channel conductance which, by restoring excitability in some fibres and increases action potential amplitude in other fibres, better maintains SR Ca^2+^ release.
12. When an exercise-induced intracellular acidosis occurs concomitantly with both raised [P_i_] and reduced trans-sarcolemmal K^+^-gradients, the SR Ca^2+^ release is likely to be better maintained. We propose that the likely dominant effect involves both [H^+^]_i_ and [P_i_]_i_ (mainly [H_2_PO_4_^−^]_i_) acting to reduce force/power via myofilament proteins since these effects occur more peripherally to SR Ca^2+^ release in the sequence of events leading to contraction.
13. Current evidence suggests that intra- or extracellular H^+^/lactate^−^ does not cause central fatigue. Small increases of interstitial [H^+^]_o_, [lactate^−^]_o_ (and [ATP]_o_) together activate group III/IV muscle afferents thereby contributing to elevated RPE, fatigue sensations, and the beneficial muscle metaboreflex. An extreme plasma acidosis may cause O_2_ desaturation of haemoglobin to provoke a severe cerebral hypoxia to lower voluntary activation from the CNS (Fig. 12).
14. Experimental modulation of muscle H^+^/lactate^−^ regulatory processes (i.e., extracellular H^+^-buffers, MCT, CA, intramuscular carnosine) provide strong evidence for a small but functionally important role of H^+^/lactate^−^ in fatigue (Fig. 6).
15. Studies on animal muscles in situ and in vitro replicate many findings with acidosis on human exercise performance, hence are valuable models to examine mechanisms for effects of acidosis on performance.

Finally, we need to address specifically when lactic-acidosis contributes to fatigue in human exercise performance. Clearly, high-intensity exercise or muscle contractions must be involved for such effects to occur. We contend that this mainly involves dynamic exercise at intensities exceeding 80% VO_2_ peak, whether continuous or repeated (intermittent) exercise, when fast-twitch motor units are recruited, and for exercise lasting 2–10 min (i.e., the time frame when the largest intracellular acidosis occurs). Lactate^−^ and acidosis are involved in fatigue of prolonged or repeated isometric contractions of >50% MVIC (with or without blood flow occlusion) when surpassing 1 min duration. We recognise that fatigue is multifactorial and depends on the characteristics of the participant, the muscles activated, and nature of the exercise regimes employed. Whilst intracellular acidosis is important in high-intensity activities, it is not the sole player in fatigue (Fig. 3). There are also contributions from central fatigue, metabolic and fuel supply changes, ionic disturbances, ROS formation, and impaired Ca^2+^ handling, regardless of whether these effects occur individually or by interacting with acidosis (Cairns 2013; Debold et al. 2016; Hostrup et al. 2021; Nybo and Rasmussen 2007).

### Summary

Our evaluation of current data helps us to understand and reconcile the views of some researchers. Intracellular acidosis to pH_i_ 6.7–6.6 per se has little or no effect on peak isometric force (Westerblad 2016). A larger acidosis to pH_i_ 6.5–6.2 per se, which occurs only in fast-twitch fibres, depresses peak force (especially at subsaturating [Ca^2+^]_i_), slows shortening velocity, and reduces muscle power (Fitts 2016) to impair exercise performance. Pre-exercise induced acidosis leads to impairment of exercise performance in simulated sports activities, especially during early stages of fatiguing exercise in humans, and with animal muscles stimulated in situ (to 5–30% initial). We interpret this and other findings to mean that raised [H^+^]_i_ contributes to fatigue (power loss), directly and through a H^+^–P_i_ interaction (partially mediated via H_2_PO_4_^−^). We have alluded to research needed to further evaluate the role of lactic acidosis in fatigue throughout the text and in Table 4.

**Table 4:** Future research directions to evaluate the role of H^+^/lactate^−^ in fatigue

More studies should measure muscle power and shortening velocity, with pH_i_, during various exercise or stimulation regimes in humans or with isolated human muscle fibres (and with induced acidosis)
More studies should focus on fast-twitch muscle/fibres where a larger intracellular acidosis occurs
Determine why different responses occur with raised [lactate^−^]_o_ on non-fatigued and fatiguing muscle, with measurement of pH_i_
Evaluate the effects of lowered pH_i_ and raised [H_2_PO_4_^−^]_i_ on muscle processes using exercise pH_i_ values (6.7–6.2) and [H_2_PO_4_^−^]_i_ of up to 25 mM, with measurement of force, velocity and power
Determine why some but not all induced acidification interventions accelerate fatigue (↓ force/power) in exercising humans or stimulated muscles/fibres
Determine how physiological pH_i_ changes alter various metabolic species, muscle cellular processes, and muscle/exercise performance
Understand mechanisms for the H^+^–P_i_ interaction on muscle function (using physiological changes) with decreased PCr, elevated ROS, and myosin-light chain phosphorylation. Determine whether synergistic or additive processes occur at the myofilament protein level
Understand processes involved in the K^+^-H^+^ interaction on force (using physiological changes) and its interaction with catecholamines, Na^+^, Ca^2+^, glycogen, and ROS in single muscle fibres
Determine whether the H^+^–P_i_ or K^+^–H^+^ effect dominates the force response by investigating combined changes of these factors
Understand how exercise-induced acidosis alters mitochondrial function
Understand any link between severe plasma acidosis, desaturation of haemoglobin, and central fatigue
More studies should focus on manipulating H^+^-buffers and H^+^/lactate^−^ regulators, to test their effects on exercise and muscle performance, together with measurement of pH_i_
Correlation studies should not be used as a measure to establish cause and effect between raised [H^+^]/[lactate^−^] and fatiguing performance

## Supplementary Information

Below is the link to the electronic supplementary material.

Supplementary file1 (PDF 110 KB)

Supplementary file2 (PDF 243 KB)

## Data Availability

Two supplementary files are now available.

## Abbreviations

ATP: Adenosine triphosphate
9-AC: 9-Anthracenecarboxylic acid
CA: Carbonic anhydrase
cAMP: Cyclic adenosine monophosphate
CICR: Calcium-induced calcium release
ClC-1: Sarcolemmal chloride channels
CNS: Central nervous system
DHPR: Dihydropyridine receptor—Voltage-sensor of T-system membranes
EDL: Extensor digitorum longus muscle
FDB: Flexor digitorum brevis muscle
GLUT4: Glucose transporter protein in skeletal muscle sarcolemma
H_2_PO_4_^−^: Diprotonated phosphate
HPO_4_^2^^−^: Monoprotonated phosphate
K_ATP_: ATP-sensitive potassium channel
[lactate^−^]_i_: Intracellular lactate concentration
[lactate^−^]_o_: Extracellular lactate concentration
MCT: Monocarboxylate (lactate) transporter
MVIC: Maximum voluntary isometric contraction
M-wave: Compound extracellular muscle action potential
NaV1.4: Voltage-activated sodium channel
NaHCO_3_: Sodium bicarbonate
NH_4_Cl: Ammonium chloride
NHE1: Sodium-hydrogen exchanger
PCr: Phosphocreatine
PDH: Pyruvate dehydrogenase
PFK: Phosphofructokinase
pH_i_: Intracellular pH
pH_o_: Extracellular pH
phos: Glycogen phosphorylase
P_i_: Total inorganic phosphate
^31^P-MRS: Phosphorus nuclear magnetic resonance spectroscopy
ROS: Reactive oxygen species
RPE: Rating of perceived exertion
RyR1: Ryanodine receptor—Ca^2+^ release channel of sarcoplasmic reticulum
S_a_O_2_: Arterial oxygen saturation of haemoglobin
SERCA: Ca^2+^-pump of sarcoplasmic reticulum
SR: Sarcoplasmic reticulum
TnC: Troponin C
TnI: Troponin I
T-system: Transverse tubular system
V_max_: Maximal muscle shortening velocity
