# Supplementary material for: Lactic acidosis: implications for human exercise performance
Source: Eur J Appl Physiol. 2025 Mar 15;125(7):1761–95. doi: 10.1007/s00421-025-05750-0 (PMC12227488; doi:10.1007/s00421-025-05750-0)
Supplement: Supplementary file 1 — Supplementary file1 (PDF 110 KB) [file 421_2025_5750_MOESM1_ESM.pdf]

Combined resting values

				[lact-]i rest	[lact-]o rest
Juel 1990	Knee extensions		3.18 min	2.9	0.5
Bangsbo 1996	Knee extensions		4.67 min	2.4	1
Pan 1991	finger flexion		3-7 min		1
Kowalchuk 2000	wrist flexion ramp		12 min		1
Nielsen 2002	forearm flexors repeated 40%MV(		5 min		1
Raymer 2004	wrist flexion progressive				1.7
Volianitis 2018	forearm flexo approx rowing				1.4
Sahlin 1975	Quads 68%MVC (cuff)			1.1	
Chasiotis 1982	Quads 68%MVC (cuff)			2	
Sahlin Henriksson 1984		Quads 61%MVC (cuff)	healthy	1.2	
			trained	1.2	
Sahlin Ren 1989	Quads, 66%MVC		52s	1.2	
Mannion 1995	Quads, 60%MVC		64 s	2.3	
Hultman 1985	quads 20Hz continuous		75	1.4	
Chastoisis 1987	quads 20 Hz intermittent		54 s	1.2	
	quads 20 Hz continuous (occluded)		52s	1.2	
Spriet 1987b	quads 20Hz for 1.6 s repeated		205 s	1.8	
Jones 2009	Tib Ant 50 Hz 1.6 s repeated (occluded)		32s	0.7	
Wilkes 1983	800m				1.21
Cheetham 1986	VL 30-s sprint			0.9	0.73
Neville 1989	30s all out			1.4	
	110% VO2max 2 min			1.3	
Medbo Sejersted 1985	treadmill	end trained	1 min		0.5
		spr trained	1 min		0.8
Greenhaff 1994	VL 30-s sprint			1.1	0.84
Krustrup 2006	VL Yo-yo	trained		1.9	
Mohr 2007	VL Yo-yo			1.9	2.4
Hanon 2010	400 m		trained		3.8
Sahlin 1976	5min 0.5 Wmax then Wmax		10-11 min		1
Sahlin 1978				1.7	1
Sharp 1986	Incremental cycling			1.47	1
Kowalchuk 1988	30s isokin sprint				1
Spriet 1989	30s isokin sprint X3				
Bogdanis 1989	30s all out			1.3	0.7
Mannion 1995	Quads, modified Wingate		30-s	2.3	
Linossier 1997	120% VO2peak			1.6	2.5

Hargreaves 1998	30s isokin sprint X3			1.7	1.2
Parolin 1999	30s isokin sprint X3	90s +		0.9	
Harmer 2000	130% VO2max			2	1
McCartney 1986	4x 30s			2.1	1
Messonnier 2007	120% VO2max				3
Gunnarsson 2013	Intense repeated				1
Blain 2016	5km time trial	8.75 min			1.2
Black 2017	severe heavy domains			2	1
Correia-Oliveira 2017	4km time trial	6 min 20s			1
Black 2018	incr				1
	intermittent 3 min x 2				1
Fiorenze 2019	intermittent sprints			0.4	
Vigh-Larsen 2022	repeated 45 s X 10			3.4	2
Mildenhall 2023	3 min of 4km time trial	3 min	sprint t		1.3
			end t		1
Bers & McKenzie 1989	2000 m	6 min			1
Nielsen 1999	2000 m sim				0.6
Nielsen 2002	2000m sim				1
Volianitis 2010	2000m sim				1
Volianitis 2011	2000m sim				1.6
Volianitis 2018	2000m sim				1.2
Boegman 2022	2000 m	6 min			2
Nielsen 2022	2000 m				0.9
				1.611935	1.245854
				0.640897	0.665853
				(0.4, 3.4)	(0.5,3.8)
				31	41

	**Running**			[lact-]i rest		[lact-]i end		[lact-]o rest		[lact-]o end	
Costill 1983	125% VO2peak gastroc					18.4					
	125% VO2peak vastis L					15.9					
	400 m gastroc									12.3	
Wilkes 1983	800m							1.21		12.62	
Cheetham 1986	VL 30-s sprint			0.9		27.3		0.73		11.6	
Neville 1989	30s all out			1.4		30.1					
	110% VO2max 2 min			1.3		9.9					
Medbo Sejersted 1985	treadmill	end trained	1 min					0.5		9.8	
		spr trained	1 min					0.8		13.6	
Greenhaff 1994	VL 30-s sprint			1.1		31.4		0.84		12.9	
Krustrup 2006	VL Yo-yo	trained		1.9		24				11.5	
Mohr 2007	VL Yo-yo			1.9		15.9		2.4		11	
Hanon 2010	400 m		trained					3.8		16.4	
				1.416667		21.6125		1.468571		12.41333	
				0.411906	n=6	7.733496	8	1.203861	7	1.868047	9

				(0.9-1.9)		(9.9-31.4)		(0.7-3.8)		(9.8-16.4)	
	**Cycling**			[lact-]i rest		[lact-]i end		[lact-]o rest		[lact-]o end	
Sahlin 1976	5min 0.5 Wmax then Wmax	10-11 min						1		11	
Sahlin 1978				1.7		32		1		18	
Sharp 1986	Incremental cycling			1.47		29.4		1		6.5	
Kowalchuk 1988	30s isokin sprint			5.5		47		1		13	
Spriet 1989	30s isokin sprint X3					31.7					
Bogdanis 1989	30s all out			1.3		41.7		0.7		16.9	
Mannion 1995	Quads, modified Wingate	30-s		2.3		34.9					
Linossier 1997	120% VO2peak			1.6		35.6		2.5		13.4	
Hargreaves 1998	30s isokin sprint X3			1.7		36.6		1.2		16.9	
Parolin 1999	30s isokin sprint X3	90s +		0.9		35					
Harmer 2000	130% VO2max			2		39.6		1		8.7	
McCartney 1986	4x 30s			2.1		51.6		1		22	
Messonnier 2007	120% VO2max					35.6		3		17.6	
Gunnarsson 2013	Intense repeated					28.9		1		13.2	
Blain 2016	5km time trial	8.75 min						1.2		12.7	
Black 2017	severe heavy domains			2		39.6		1		7.5	
Correia-Oliveira 2017	4km time trial	6 min 20s						1		8	
Black 2018	incr							1		7	
	intermittent 3 min x 2							1		7	
Fiorenze 2019	intermittent sprints			0.4		28					
Vigh-Larsen 2022	repeated 45 s X 10			3.4		27.2		2		12.7	
Mildenhall 2023	3 min of 4km time trial	3 min	sprint t					1.3		17.5	
			end t					1		21.7	
				2.028462		35.9		1.257895		13.22632	
				1.264264	13	6.808818	16	0.589082	19	4.97168	19
				(0.4-5.5)		(27.2-51.6)		(1-2.5)		(7.0-22.0)	
	**rowing**			[lact-]i rest		[lact-]i end		[lact-]o rest		[lact-]o end	
Bers & McKenzie 1989	2000 m	6 min						1		17.9	
Nielsen 1999	2000 m sim							0.6		26.2	
Nielsen 2002	2000m sim							1		16.2	
Volianitis 2010	2000m sim							1		16.8	
Volianitis 2011	2000m sim							1.6		21.4	
Volianitis 2018	2000m sim							1.2		20	
Boegman 2022	2000 m	6 min						2		20	
Nielsen 2022	2000 m							0.9		18.3	
								1.1625		19.6	
								0.440576	8	3.187027	
								(0.6-2)		(16.2-26.2)	

	**Repeated Leg contractions (dynamic)**			[lact-]i rest		[lact-]i end		[lact-]o rest		[lact-]o end	
Juel 1990	Knee extensions	3.18 min		2.9		41		0.5		14.1	
Bangsbo 1996	Knee extensions	4.67 min		2.4		39		1		10	
Juel 2004	Incremental knee extensions	8.2 min	untrained			33.8		0.8		8	
			trained			20.7		0.8		10.7	
				2.65		33.625		0.775		10.7	
						9.135416	n=4	0.206155		2.539029	n=4
				(2.4, 2.9)		(20.7-41)		(0.5-1.0)		(8-14.1)	

	**Repeated Contractions Arm models**		[lact-]i rest	[lact-]i end	[lact-]o rest		[lact-]o end
Pan 1991	finger flexion	3-7 min			1		5
Kowalchuk 2000	wrist flexion ramp	12 min			1		5
Nielsen 2002	forearm flexors repeated 40%MV(	5 min			1		4.9
Raymer 2004	wrist flexion progressive				1.7		7.1
Volianitis 2018	forearm flexo approx rowing				1.4		6.8
					1.22		5.76
					0.319374	n=5	1.092245
					(1-1.7)		(5-7.1)

	**Prolonged static legs**			
Sahlin 1975	Quads 68%MVC (cuff)		1.1		37.3
Chasiotis 1982	Quads 68%MVC (cuff)		2		34.8
Sahlin Henriksson 1984	Quads 61%MVC (cuff)	healthy	1.2		29.4
		trained	1.2		20.7
Sahlin Ren 1989	Quads, 66%MVC	52s	1.2		33.3
Mannion 1995	Quads, 60%MVC	64 s	2.3		20.3
			1.5		29.3
			0.513809	n=6	7.283131
			(1.1-2.3)		(20.3-37.3)

	**stim contract (human)**				
Hultman 1985	quads 20Hz continuous	75	1.4		26.4
Chastoisis 1987	quads 20 Hz intermittent	54 s	1.2		39.6
	quads 20 Hz continuous (occluded)	52s	1.2		28.6
Spriet 1987b	quads 20Hz for 1.6 s repeated	205 s	1.8		50.9
Constantin-Teodosiu :	Tib Ant 50 Hz 1.6 s repeated				
Jones 2009	Tib Ant 50 Hz 1.6 s repeated (occluded)	32s	0.7		28
			1.26		34.7
			0.397492	n=5	10.45753

(0.7-1.8)	(26.4-50.9)
