# Supplementary material for: Lactic acidosis: implications for human exercise performance
Source: Eur J Appl Physiol. 2025 Mar 15;125(7):1761–95. doi: 10.1007/s00421-025-05750-0 (PMC12227488; doi:10.1007/s00421-025-05750-0)
Supplement: Supplementary file 2 — Supplementary file2 (PDF 243 KB) [file 421_2025_5750_MOESM2_ESM.pdf]

Combined resting values

				pHi rest	[H+]i rest	pHo rest
Juel 1990	Knee extensions	3.18 min		7.14	72	7.4
Bangsbo 1996	Knee extensions	4.67 min		7.17	68	
Juel 2004	Incremental knee extensions	8.2 min	untrained	7.16	69	7.37
			trained	7.15	71	7.38
Burnley 2010	Quads repeated isometric maxima	5 min		7.02	95	
	Quads repeated isometric 54%MVC to task fail				95	
Layec 2013	Quads, ext every 3 s for 1 min	3-6 min		7.04	91	
Broxterman 2017a	Quads, 3s on 2s off MVC	5 min		7	100	
Broxterman 2017b	Quads, 3s on 2s off 58% MVC	5 min		7.01		
Sundberg 2019	Kicking ever 2s for 4 min	4 min	young	7	100	
			old	7	100	
Bartlett 2020	Quads, max dynamic every 2s	4 min		7	100	
Hureau 2022	Quads, 3s on 2s off 5 min	5 min		7	100	
Fitzgerald 2023	Quads, isotonic/kinetic	4 min	young	7	100	
			older	7	100	
	**Calf/Tib anterior**					
Weiner 1990	tib ant every 10s	5 min		7.07	85	
Raymer 2007	plantar-flexion incremental	14 min		6.99	102	
Zange 2008	calf repeated intermit 80% max pc	10 min		7.04	100	
	15-s contract, 45s rest					
Churchwall 2010	planta flexion mod to heavy			6.98	105	
Layec 2013	plantar flex isokinet every 2,5s	3-8 min		7.02	95	
Moll 2016	plantar flex isokinet every 2,5s	3 min		7.03	93	
	**Prolonged static legs**					
Sahlin 1975	Quads 68%MVC (cuff)	3 min		7.09	81	
Chasiotis 1982	Quads 68%MVC (cuff)			7	100	
Sahlin Henriksson 1984	Quads 61%MVC (cuff)	healthy		7.12	76	
		trained		7.1	79	
Weiner 1990	TA sustained	4 min		7.03	93	
		trained		7.1	79	
Mannion 1995	Quads, 60%MVC	64 s		7.17	68	
DeGroot 1993	plantar flexors sustained MVC (gastroc)			7.09	81	
Kent-Braun 1999	dorsiflexors sustained MVC	4 min		7.01	98	
	**Running Leg contractions**					
Costill 1983	125% VO2peak gastroc	82 s		7.032	93	
	125% VO2peak vastis L			7.036	92	7.35
	400 m gastroc			7.03	93	
Wilkes 1983	800m					7.4
Cheetham 1986	VL 30-s sprint			7.05	89	7.4
Mohr 2007	VL Yo-yo			7.02	95	
Neville 1989	30s all out			7.02	95	7.38
	110% VO2max 2 min			7.03	93	7.39
Medbo Sejersted 1985	treadmill end trained	1 min				7.43
	spr trained	1 min				7.41
Greenhaff 1994	VL 30-s sprint					7.38
Krustrup 2006	VL Yo-yo trained			7.07	85	
Hanon 2010	400 m trained					7.39
	**Cycling Leg contractions**					
Hermanssen Osnes	Quads maximal	2 min		6.92	120	7.42

Sahlin 1976	5min 0.5 Wmax then Wmax	10-11 min		7.08	83	7.45
Sahlin 1978	5min 0.5 Wmax then Wmax	10.5 min		7	100	7.38
Sharp 1986	Incremental cycling			7.08	83	7.38
Kowalchuk 1988	30s isokin sprint	30 s		6.88	132	7.38
Spriet 1989	30s isokin sprint X3					
Bogdanis 1989	30s all out	30 s		7.16	69	7.38
Mannion 1995	Quads, modified Wingate	30-s		7.17	68	
Linossier 1997	120% VO2peak	5 min		7.16	69	7.4
Hargreaves 1998	30s isokin sprint X3			7.17	69	7.38
Parolin 1999	30s isokin sprint X3			7.21	62	
Harmer 2000	130% VO2max			7.2	63	7.4
Messonnier 2007	120% VO2max			7.16	69	7.42
Gunnarsson 2013	Intense repeated			7.13	74	
Blain 2016	5km time trial	8.75 min		6.91	123	7.35
Vantaholo 2016	3-min all out	3 min		7.3	50	
Black 2017	severe heavy domains			7.03	93	
Correia-Oliveira 2017	4km time trial	6min 20s				7.4
Black 2018	intermitt 3 min x 2			7.04	91	
	incremental			7.03	93	
Fiorenze 2019	intermittent sprints			7.27	54	
Gough 2019	4km time trial					7.4
Vigh-Larsen 2022	repeated 45 s X 10, 105%Vo2max			7.01	98	
Mildenhall 2023	3 min of 4km time trial	3 min	sprint t			7.35
			end t			7.35
	**Rowing arm contractions**					
Nielsen 1999	2000 m sim					7.4
Nielsen 2002	2000m sim	6 min 30				7.4
Volianitis 2010	Wrist 2000m sim					7.43
Volianitis 2011	2000m sim					7.41
Volianitis 2018	Wrist 2000m sim					7.36
Boegman 2022	2000 m					7.41
Nielsen 2022	2000 m					7.42
	**Stimulated isometric contractions (humans)**					
Hultman 1985	quads 20Hz continuous	75 s		7.04	89	
Chastoisis 1987	quads 20 Hz intermittent	54 s		7.09	81	
	quads 20 Hz continuous (occluded)	52s		7.09	81	
Spriet 1987a	quads 20Hz for 1.6 s repeated (occluded)	205 s		7	100	
Constantin-Teodosiu 1	Tib Ant 50 Hz 1.6 s repeated	32s		7.06	87	
Jones 2009	Tib Ant 50 Hz 1.6 s repeated (occluded)	70s		7.01	98	
	**Repeated arm contractions (dynamic or isometric) Finger, wrist, forarm**					
Taylor 1983	Squeezing every 2-s (finger flexor)	20 min		7.03	93	
Miller 1988	AP repeat 75% MVC	45 min		7.08	83	
Hood 1988	Forearm 2 min	2 min		7.06	87	
Wilson 1988	Max Wrist flex for 1s every 5s	4 min		7.01	98	
	Max Wrist flex for 2s every 5s	4 min		7	100	
Newham 1990	FDI every 2s for 10 min (50%MVC)	10 min		7.1	79	
	FDI every 2s for 10 min (100%MVC)			7.1	79	

Weiner 1990	AP		7.03	93	
Kemp 1993	flexor dig super repeated handgrip		7	100	
Bouchel 1998	repeated handgrip 30%MVC (flexor digitorum superficialis)	2 min	7	100	7.39
Kowalchuk 2000	wrist flexion ramp	12 min	7.02	100	7.41
Nielsen 2002	forearm flexors repeated 40%MVC	5 min	7.06	87	7.43
Raymer 2004	wrist flexion progressive for 1s every 2 s		7.08	83	7.43
Raymer 2009	wrist extension progressive (extensor carpi radialis breavis)		7.03	93	7.43
Layec 2013	finger flexors 15%MVC	3 min	7.03	93	
Volianitis 2018	forearm flex Handgrip				7.39
			7.05959	88.4359	7.39575
			0.074915	14.50624	0.024795
			(6.88, 7.30)	(50-132)	(7.35, 7.45)
			78	78	40

	**Running Leg contractions**			pHi rest		pHi end	[H+]i rest	[H+] end	pHo rest		pHo rend
Costill 1983	125% VO2peak gastroc		82 s	7.032		6.88	93	132			
	125% VO2peak vastis L			7.036		6.856	92	139	7.35		7.1
	400 m gastroc			7.03		6.63	93	234			7.1
Wilkes 1983	800m								7.4		7.07
Cheetham 1986	VL 30-s sprint			7.05		6.73	89	186	7.4		7.16
Mohr 2007	VL Yo-yo			7.02		6.79	95	162			
Neville 1989	30s all out			7.02		6.78	95	166	7.38		7.17
	110% VO2max 2 min			7.03		6.92	93	120	7.39		7.25
Medbo Sejersted 1985	treadmill	end trained	1 min						7.43		7.25
		spr trained	1 min						7.41		7.16
Greenhaff 1994	VL 30-s sprint								7.38		7.01
Krustrup 2006	VL Yo-yo	trained		7.07		6.8	85	158			
Hanon 2010	400 m	trained							7.39		7.08
									7.37		7.04
				7.036		6.79825	91.875	162.125	7.392222		7.135
				0.016699	8	0.091192	3.356763	35.82672	0.022236	9	0.077639
				(7.02-7.07)		(6.63-6.92)	(85-95)	(120-234)	(7.35-7.43)		(7.07-7.25)

	**Cycling Leg contractions**		pHi rest	pHi end	[H+]i rest	[H+] end	pHo rest	pHo rend
Hermanssen Osnes	Quads maximal	2 min	6.92	6.41	120	389	7.42	7.11
Sahlin 1976	5min 0.5 Wmax then Wmax	10-11 min	7.08	6.6	83	251	7.45	7.2
Sahlin 1978	5min 0.5 Wmax then Wmax	10.5 min	7	6.4	100	398	7.38	6.98
Sharp 1986	Incremental cycling		7.08	6.64	83	229	7.38	6.98
Kowalchuk 1988	30s isokin sprint	30 s	6.88	6.48	132	328	7.38	7
Spriet 1989	30s isokin sprint X3			6.5		315		
Bogdanis 1995	30s all out	30 s	7.16	6.72	69	223	7.38	7.1
Mannion 1995	Quads, modified Wingate	30-s	7.17	6.68	68	209		
Linossier 1997	120% VO2peak	5 min	7.16	6.49	69	324	7.4	7.21
Hargreaves 1998	30s isokin sprint X3		7.17	6.65	69	191	7.38	7.1
Parolin 1999	30s isokin sprint X3		7.21	6.59	62	255		
Harmer 2000	130% VO2max		7.2	6.57	63	270	7.4	7.25
Messonnier 2007	120% VO2max		7.16	6.49	69	324	7.42	7.21
Gunnarsson 2013	Intense repeated		7.13	6.81	74	155		
Blain 2016	5km time trial	8.75 min	6.91	6.69	123	204	7.35	7.15
Vanhatolo 2016	3-min all out	3 min	7.3	6.65	50	224		
Black 2017	severe heavy domains		7.03	6.52	93	302		
Correia-Oliveira 2017	4km time trial	6min 20s					7.4	7.2

Black 2018	intermitt 3 min x 2			7.04		6.65		91	224			
	incremental			7.03		6.77		93	170			
Fiorenze 2019	intermittent sprints			7.27		6.74		54	182			
Gough 2019	4km time trial									7.4		7.2
Vigh-Larsen 2022	repeated 45 s X 10, 105%Vo2max			7.01		6.66		98	219			
Mildenhall 2023	3 min of 4km time trial	3 min	sprint t							7.35		7.21
			end t							7.35		7.05
				7.0955		6.605238		83.15	256.4762	7.389333		7.13
				0.11727	20	0.115266	21	22.95138	69.11774	0.028149	15	0.092273
				(6.88-7.3)		(6.40-6.81)		(74-100)	(155-398)	(7.35-7.45)		(6.98-7.25)

	Rowing arm contractions			pHi rest		pHi end		[H+]i rest	[H+] end	pHo rest		pHo rend
Nielsen 1999	2000 m sim									7.4		6.85
Nielsen 2002	2000m sim	6 min 30								7.4		7.05
Volianitis 2010	Wrist 2000m sim			7.05		6.32		89	479	7.43		6.9
Volianitis 2011	2000m sim									7.41		7.02
Volianitis 2018	Wrist 2000m sim			7		6.3		100	501	7.36		6.95
Boegman 2022	2000 m									7.41		7.2
Nielsen 2022	2000 m									7.42		7.17
				7.025		6.31		94.5	490			
						0.014142	n=2		15.55635	7.404286		7.02
						(6.30-6.32)			(479, 501)	0.022254	7	0.131656
										(7.36-7.43)		(6.85=7.2)

	Cycling Leg contractions			pHi rest		pHi end		[H+]i rest	[H+] end	pHo rest		pHo rend
Hermanssen Osnes	Quads maximal	2 min		6.92		6.41		120	389	7.42		7.11
Sahlin 1976	5min 0.5 Wmax then Wmax	10-11 min		7.08		6.6		83	251	7.45		7.2
Sahlin 1978	5min 0.5 Wmax then Wmax	10.5 min		7		6.4		100	398	7.38		6.98
Sharp 1986	Incremental cycling			7.08		6.64		83	229	7.38		6.98
Kowalchuk 1988	30s isokin sprint	30 s		6.88		6.48		132	328	7.38		7
Spriet 1989	30s isokin sprint X3					6.5			315			
Bogdanis 1995	30s all out	30 s		7.16		6.72		69	223	7.38		7.1
Mannion 1995	Quads, modified Wingate	30-s		7.17		6.68		68	209			
Linossier 1997	120% VO2peak	5 min		7.16		6.49		69	324	7.4		7.21
Hargreaves 1998	30s isokin sprint X3			7.17		6.65		69	191	7.38		7.1
Parolin 1999	30s isokin sprint X3			7.21		6.59		62	255			
Harmer 2000	130% VO2max			7.2		6.57		63	270	7.4		7.25
Messonnier 2007	120% VO2max			7.16		6.49		69	324	7.42		7.21
Gunnarsson 2013	Intense repeated			7.13		6.81		74	155			
Blain 2016	5km time trial	8.75 min		6.91		6.69		123	204	7.35		7.15
Vanhatolo 2016	3-min all out	3 min		7.3		6.65		50	224			
Black 2017	severe heavy domains			7.03		6.52		93	302			
Correia-Oliveira 2017	4km time trial	6min 20s								7.4		7.2
Black 2018	intermitt 3 min x 2			7.04		6.65		91	224			
	incremental			7.03		6.77		93	170			
Fiorenze 2019	intermittent sprints			7.27		6.74		54	182			
Gough 2019	4km time trial									7.4		7.2
Vigh-Larsen 2022	repeated 45 s X 10, 105%Vo2max			7.01		6.66		98	219			
Mildenhall 2023	3 min of 4km time trial	3 min	sprint t							7.35		7.21
			end t							7.35		7.05

			7.0955		6.605238		83.15	256.4762	7.389333		7.13
			0.11727	20	0.115266	21	22.95138	69.11774	0.028149	15	0.092273
			(6.88-7.3)		(6.40-6.81)		(74-100)	(155-398)	(7.35-7.45)		(6.98-7.25)

	**Rowing arm contractions**		pHi rest		pHi end		[H+]i rest	[H+] end	pHo rest		pHo rend
Nielsen 1999	2000 m sim								7.4		6.85
Nielsen 2002	2000m sim	6 min 30							7.4		7.05
Volianitis 2010	Wrist 2000m sim		7.05		6.32		89	479	7.43		6.9
Volianitis 2011	2000m sim								7.41		7.02
Volianitis 2018	Wrist 2000m sim		7		6.3		100	501	7.36		6.95
Boegman 2022	2000 m								7.41		7.2
Nielsen 2022	2000 m								7.42		7.17
			7.025		6.31		94.5	490			
					0.014142	n=2		15.55635	7.404286		7.02
					(6.30-6.32)			(479, 501)	0.022254	7	0.131656
									(7.36-7.43)		(6.85=7.2)

Repeated Leg contractions (dynamic or isometric)

Quads

				pHi rest		pHi end		[H+]I rest		[H+] end		pHo rest	pHo rend		
Juel 1990	Knee extensions	3.18 min		7.14		6.71		72		195		7.4	7.1		
Bangsbo 1996	Knee extensions	4.67 min		7.17		6.82		68		153					
Juel 2004	Incremental knee extensions	8.2 min	untrained	7.16		6.69		69		204		7.37	7.13		
			trained	7.15		6.82		71		151		7.38	7.07		
Burnley 2010	Quads repeated isometric maxima	5 min		7.02		6.64		95		229					
	Quads repeated isometric 54%MVC to task fail					6.69				204					
												7.383333	7.1	7.1	
Layec 2013	Quads, ext every 3 s for 1 min	3-6 min		7.04		6.9		91		125				0.03	n=3
														(7.07-7.13)	
Broxterman 2017a	Quads, 3s on 2s off MVC	5 min		7		6.54		100		288					
Broxterman 2017b	Quads, 3s on 2s off 58% MVC	5 min		7.01		6.54		98		288					
Sundberg 2019	Kicking ever 2s for 4 min	4 min	young	7		6.73		100		186					
			old	7		6.61		100		245					
Bartlett 2020	Quads, max dynamic every 2s	4 min		7		6.6		100		251					
Hureau 2022	Quads, 3s on 2s off 5 min	5 min		7		6.5		100		316					
Fitzgerald 2023	Quads, isotonic/kinetic	4 min	young	7		6.46		100		355					
			older	7		6.54		100		288					

Calf/Tib anterior

Weiner 1990	tib ant every 10s	5 min	7.07	6.73	85	186
Raymer 2007	plantar-flexion incremental	14 min	6.99	6.57	102	269
Zange 2008	calf repeated intermit 80% max pc	10 min	7.04	6.4	100	398
	15-s contract, 45s rest					
Churchwall 2010	planta flexion mod to heavy		6.98	6.7	105	200
Layec 2013	plantar flex isokinet every 2,5s	3-8 min	7.02	6.73	95	186
Moll 2016	plantar flex isokinet every 2,5s	3 min	7.03	6.45	93	355

calf	7.021667		6.596667		96.66667		265.6667	
	0.033116	n=6	0.146379	6	7.229569	6	92.20557	6
	(6.98-7.07)		(6.4-6.73)		(85-105)		(186-398)	
Quads	7.049286		6.652667		90.28571		231.8667	
	0.070543	14	0.128867	15	13.58409	14	66.24399	15
	(7.0-7.17)		(6.46-6.90)		(68-100)		(125-355)	
all leg	7.041		6.636667		92.2		241.5238	
	0.062146	20	0.132866	21	12.20699		73.77033	21
	(6.98-7.17)		(6.4-6.90)		(68-105)		(125-398)	

Repeated arm contractions (dynamic or isometric)
Finger, wrist, forarm

			pHi rest	pHi end	[H+]i rest	[H+]i end
Taylor 1983	Squeezing every 2-s	20 min	7.03	6.38	93	417
	(finger flexor)					
Miller 1988	AP repeat 75% MVC	45 min	7.08	6.55	83	281
Hood 1988	Forearm 2 min	2 min	7.06	6.31	87	490
Wilson 1988	Max Wrist flex for 1s every 5s	4 min	7.01	6.24	98	575
	Max Wrist flex for 2s every 5s	4 min	7	5.86	100	1380
Newham 1990	FDI every 2s for 10 min (50%MVC)	10 min	7.1	6.6	79	251
	FDI every 2s for 10 min (100%MVC)		7.1	6.61	79	245
Weiner 1990	AP		7.03	6.55	93	281
Kemp 1993	flexor dig super repeated handgrip		7	6.05	100	891
				6.14		724
				6.3		501
				6.4		398
				6.55		282
Bouchel 1998	repeated handgrip 30%MVC	2 min	7	6.33	100	468
	(flexor digitorum superficialis)					
Kowalchuk 2000	wrist flexion ramp	12 min	7.02	6.43	100	371

Nielsen 2002	forearm flexors repeated 40%MVC	5 min	7.06		6.36		87	437	
Raymer 2004	wrist flexion progressive for 1s every 2 s		7.08		6.35		83	447	
Raymer 2009	wrist extension progressive (extensor carpi radialis breavis)		7.03		6.52		93	302	
Layec 2013	finger flexors 15%MVC	3 min	7.03		6.58		93	263	
Volianitis 2018	forearm flex Handgrip				6.5			316	
			7.042		6.3805		91.2	466	
			0.035496	15	0.196883	20	7.72935	271.5937	20
			(6.98-7.1)		(5.86-6.61)		(79-105)	(245-1202)	

Prolonged static legs

		pHi rest		pHi end	[H+]I rest	[H+] end
Quads 68%MVC (cuff)	3 min	7.09		6.56	81	275
Quads 68%MVC (cuff)		7		6.6	100	251
Quads 61%MVC (cuff)	healthy	7.12		6.61	76	245
	trained	7.1		6.8	79	158
TA sustained	4 min	7.03		6.52	93	302
	trained	7.1		6.8	79	158
Quads, 60%MVC	64 s	7.17		6.89	68	129
plantar flexors sustained MVC (gastroc)		7.09		6.47	81	339
dorsiflexors sustained MVC	4 min	7.01		6.49	98	324
		7.078889		6.637778	83.88889	242.3333
		0.055327	9	0.153442	10.72898	77.29166
		(7-0.17)		(6.47-6.89)	(68-100)	(129-339)

	**Prolonged static arms**		pHi rest		pHi end		[H+]I rest	[H+] end
Miller 1987	AP sustained MVC	4 min	7.1		6.4		79	398
Miller 1988	AP sustained MVC	4 min	7.08		6.58		83	263
Weiner 1990	AP sustained	4 min	7.03		6.34		93	457
Cady 1989	FDI 15sMVC 3X ischaemia		7.03		6.51		93	309
			7.06		6.4575		87	356.75
			0.03559	n=4	0.107819		7.118052	87.21764
					(6.34-6.58)		(79-93)	(263-457)

Stimulated isometric contractions (humans)

			pHi rest		pHi end		[H+]I rest	[H+] end
Hultman 1985	quads 20Hz continuous	75 s	7.04		6.7		89	200
Chastoisis 1987	quads 20 Hz intermittent	54 s	7.09		6.53		81	297
	quads 20 Hz continuous (occluded)	52s	7.09		6.66		81	221
Spriet 1987a	quads 20Hz for 1.6 s repeated (occluded)	205 s	7		6.43		100	372
Constantin-Teodosiu 1	Tib Ant 50 Hz 1.6 s repeated	32s	7.06		6.66		87	219
Jones 2009	Tib Ant 50 Hz 1.6 s repeated (occluded)	70s	7.01		6.65		98	224
			7.048333		6.605		89.33333	255.5
			0.038687	n=6	0.103296	n=6	8.164966	66.1657
			(7.0-7.09)		(6.43-6.7)		(81-100)	(200-372)
